# A Synthetic Lethality-Informed Multi-Omic Framework for Identifying a Five-Gene Diagnostic Signature in Chronic Obstructive Pulmonary Disease

**DOI:** 10.3390/cimb48050475

**Published:** 2026-05-02

**Authors:** Yue Yang, Zengrui Wang, Xiaorong Su, Jiefu Tang, Zhi Zhang, Xinli Fan, Haitao Xu, Lihan Wang, Zhuang Luo

**Affiliations:** The First School of Clinical Medicine, Kunming Medical University, Kunming 650500, China; 2024542084@kmmu.edu.cn (Y.Y.); 20240467@kmmu.edu.cn (Z.W.); 20240623@kmmu.edu.cn (X.S.); 20210679@kmmu.edu.cn (J.T.); 20230593@kmmu.edu.cn (Z.Z.); 20230423@kmmu.edu.cn (X.F.); 2025504023@kmmu.edu.cn (H.X.); 2024501307@kmmu.edu.cn (L.W.)

**Keywords:** chronic obstructive pulmonary disease, synthetic lethality, machine learning, biomarkers, immune microenvironment, molecular docking

## Abstract

Chronic obstructive pulmonary disease (COPD) lacks reliable molecular biomarkers for early diagnosis and risk stratification beyond conventional spirometry-based assessment. Synthetic lethality (SL)-related gene prioritization provides a biologically informed framework for identifying disease-associated candidate biomarkers in COPD. In this study, we integrated public transcriptomic datasets, SL-related gene sets, and machine learning approaches to identify a diagnostic signature for COPD. Using GSE47460 as the training cohort (220 COPD and 108 controls) and GSE57148 as the external validation cohort (98 COPD and 91 controls), we identified 74 SL-related differentially expressed genes enriched in inflammatory signaling and extracellular matrix organization. LASSO regression and random forest analysis yielded a five-gene diagnostic signature consisting of CYP1B1, VEGFA, RET, FGG, and S100A9. The integrated nomogram showed good diagnostic performance in the validation cohort, with an AUC of 0.8311 (95% CI: 0.7839–0.8783), outperforming individual genes and supporting its potential use as an adjunctive molecular tool for COPD diagnosis and risk assessment. Single-cell RNA sequencing, immune infiltration analysis, and preliminary in vitro experiments further supported the biological relevance of the identified genes. Overall, this study supports SL-related gene prioritization combined with multi-omic integration as a useful strategy for COPD biomarker discovery while generating testable hypotheses regarding disease-associated vulnerability pathways.

## 1. Introduction

Chronic obstructive pulmonary disease (COPD) represents one of the most formidable challenges to global public health in the 21st century. Characterized by persistent respiratory symptoms and airflow limitation, COPD is currently the third leading cause of death worldwide, imposing a staggering economic and social burden [1]. The pathogenesis of COPD is highly complex and heterogeneous, involving chronic airway inflammation, oxidative stress, protease–antiprotease imbalance, and accelerated cellular senescence [2]. Despite decades of research, the therapeutic landscape for COPD remains largely restricted to symptomatic management with bronchodilators and corticosteroids, which do not fundamentally alter the progressive decline in lung function or the high mortality associated with acute exacerbations [3]. Therefore, identifying novel diagnostic biomarkers and therapeutic targets based on a deep understanding of cell-survival mechanisms is an urgent priority.

A promising yet under-explored avenue in chronic respiratory diseases is the concept of synthetic lethality (SL). Originally conceptualized in the field of genetics and later revolutionized in oncology, synthetic lethality occurs when the simultaneous deficiency or inhibition of two genes results in cell death, whereas the deficiency of either gene alone is non-lethal [4]. In cancer research, targeting SL partners has led to the development of breakthrough therapies, such as PARP inhibitors [5]. However, the application of SL extends beyond malignant transformation. In the context of COPD, the pulmonary microenvironment is subjected to chronic environmental insults—most notably cigarette smoke and pollutants—which induce persistent DNA damage and metabolic reprogramming [6]. We hypothesized that chronic smoke- and inflammation-associated stress in COPD may enrich for transcriptional programs overlapping with genes previously implicated in synthetic lethality-related contexts, thereby offering a useful prioritization framework for biomarker discovery [7]. By intersecting the differentially expressed genes (DEGs) of COPD with established SL-related gene sets, we sought to prioritize candidate genes associated with disease stress and potential context-dependent vulnerability, rather than to identify validated synthetic lethal pairs.

In the present study, we integrated bulk transcriptomic data with SL-related gene sets to identify a novel five-gene diagnostic signature—consisting of CYP1B1, VEGFA, RET, FGG, and S100A9—using LASSO and random forest machine learning algorithms [8,9]. We further validated this signature across external cohorts and utilized single-cell RNA sequencing (scRNA-seq) to delineate the cell type-specific expression patterns of these markers within the COPD microenvironment, focusing on alveolar types and smooth muscle cells [10,11]. Finally, through drug repurposing analysis and molecular docking, we prioritized Rutin and other small molecules as candidate compounds for future functional validation [12,13]. In current clinical practice, spirometry-based indices such as FEV1 and the FEV1/FVC ratio remain central to COPD diagnosis and staging; however, these physiological measures do not fully reflect the molecular heterogeneity of the disease, particularly in early or biologically active stages. In addition, circulating inflammatory markers such as C-reactive protein (CRP) have shown associations with systemic inflammation in COPD, but their disease specificity and diagnostic stability remain limited. Therefore, a multi-gene molecular signature may provide complementary information beyond conventional physiological and inflammatory indicators and may help improve the biological characterization of COPD. The aim of this study was to determine whether SL-related gene prioritization, combined with multi-omic integration and machine learning, could yield a reproducible diagnostic signature and biologically plausible hypotheses in COPD. Although spirometry remains the cornerstone of COPD diagnosis, it does not fully capture early molecular alterations or the biological heterogeneity underlying disease progression. Therefore, there is a continued need for adjunctive molecular biomarkers that can complement conventional physiological assessment and improve risk stratification.

## 2. Materials and Methods

### 2.1. Data Acquisition

In this study, both bulk and single-cell transcriptomic datasets of human lung tissues were systematically acquired from the publicly available National Center for Biotechnology Information Gene Expression Omnibus (NCBI-GEO) database (https://www.ncbi.nlm.nih.gov/geo/ (accessed on 3 February 2026)). The GSE47460 dataset was utilized as the discovery cohort. This microarray dataset contains gene expression profiles derived from flash-frozen human whole lung homogenates, comprising 220 subjects with COPD and 108 control subjects without chronic lung disease (subjects with interstitial lung disease from the original design were excluded from our analysis) [14].

For the external validation of the machine learning model, the GSE57148 dataset was employed [15]. This independent cohort contains high-throughput RNA sequencing (RNA-seq) data generated via the Illumina HiSeq 2000 platform, consisting of lung tissue samples from 98 COPD patients and 91 control subjects.

In addition, GSE20257 was included as a supplementary external cohort for further evaluation of the diagnostic signature. This dataset contains airway tissue samples from 23 patients with COPD and 112 control subjects.

To further investigate disease pathogenesis and the cell type-specific expression patterns of the identified biomarkers at a single-cell resolution, the single-cell RNA sequencing (scRNA-seq) dataset GSE173896 was incorporated [16]. This dataset profiles the single-cell transcriptomes of human lung tissues obtained from 9 COPD patients and 7 control subjects (comprising 4 non-COPD smokers and 3 never-smokers).

Clinical characteristics available from the GEO records, including age, sex, smoking status, and lung function parameters, were manually extracted and are summarized in Table 1 where they are available. Because the completeness and annotation format of public clinical metadata differed across datasets, a fully harmonized GOLD-stage classification could not be established. In particular, FEV1/FVC data were incomplete in several cohorts; therefore, FEV1% predicted was summarized as a surrogate indicator of airflow limitation severity when available.

All data used in this study were obtained from publicly available, de-identified GEO datasets; therefore, no additional institutional ethical approval or informed consent was required for this secondary analysis.

### 2.2. Bulk Transcriptomic Data Processing

For bulk transcriptomic analyses, the discovery cohort (GSE47460) and the external validation cohort (GSE57148) were processed and analyzed separately because they were generated from different platforms. No direct merging of the two datasets was performed before differential expression analysis, feature selection, or model construction. GSE47460 was used as the discovery/training cohort, whereas GSE57148 was used exclusively as an independent external validation cohort. In the validation cohort, the processed expression matrix was based on log2-transformed TPM values. Therefore, no cross-platform batch correction or joint normalization was applied between the two cohorts. Cross-cohort comparisons were therefore based on expression trends and diagnostic performance rather than direct comparison of absolute expression values across platforms. As an additional supplementary cohort, GSE20257 was analyzed separately from both GSE47460 and GSE57148. The expression matrix of GSE20257 was processed independently, and log2(x + 1) transformation was applied before downstream analysis.

### 2.3. Identification of Differentially Expressed Genes (DEGs)

To identify DEGs between COPD patients and healthy controls in the discovery cohort (GSE47460), the limma package v.3.6 was applied [17]. A linear model was fitted, and empirical Bayes moderation was used to enhance statistical robustness. Multiple testing was controlled via the Benjamini–Hochberg procedure. Genes with an adjusted *p*-value < 0.05 and |log_2_FC| > 0.5 were defined as significant DEGs [18]. The |log_2_FC| > 0.5 threshold was adopted to capture genes with moderate but potentially meaningful biological effects while avoiding excessive exclusion of COPD-related signals in heterogeneous bulk lung tissue. This criterion also allowed adequate feature retention for downstream SL-related prioritization and machine learning analysis.

### 2.4. Identification of SL-Related Candidate Genes in COPD

To prioritize COPD-associated genes overlapping with a curated SL-related gene (SLGs) set between disease and healthy controls, SLGs were initially retrieved from the GeneCards database (https://www.genecards.org/) [19]. Genes with a relevance score greater than the median (nearly 1.22) (Supplementary Table S1) were selected, resulting in 4841 SLGs for further analysis. Subsequently, a Venn diagram was constructed using Venny 2.1 [20] to identify overlapping genes between the previously identified DEGs and the screened SLGs which finally classified in differentially expressed synthetic lethality-related genes (DESLGs). This step was designed to enrich for genes with prior relevance to synthetic lethality-related biology, rather than to infer or validate synthetic lethal interactions in COPD tissues.

### 2.5. Primary Functional Analysis of DESLGs

To comprehensively characterize the biological functions and interactions of the identified DESLGs, functional enrichment analysis was performed. Gene Ontology (GO) annotation and Kyoto Encyclopedia of Genes and Genomes (KEGG) pathway enrichment were conducted using the clusterProfiler and org.Hs.eg.db packages in R [21]. GO terms spanning biological process (BPs), cellular component (CC), and molecular function (MF), as well as KEGG pathways with an adjusted *p*-value < 0.05, were considered statistically significant [22]. The visualization of enrichment results was achieved using the ggplot2 package. Furthermore, to elucidate the protein–protein interaction (PPI) network among these genes, the gene list was submitted to the STRING database (https://string-db.org/) with a minimum required interaction score of 0.4. The resulting interaction data were imported into Cytoscape (version 3.10.2) for network construction [23].

### 2.6. Biomarker Selection Based on Machine Learning

To identify robust diagnostic biomarkers, feature selection was performed in the training cohort (GSE47460) using LASSO logistic regression and random forest analysis in R (version 4.5.1) [24]. LASSO regression was implemented with the glmnet package (version 4.1.10) using a binomial model and L1 regularization (alpha = 1), with 10-fold cross-validation and binomial deviance as the tuning metric. To reduce overfitting, the 1-standard-error criterion was adopted, yielding lambda.min = 0.0132 and lambda.1se = 0.0541 [25]; based on lambda.1se, 15 genes were retained as candidate features, including S100A9 and VEGFA. In parallel, random forest analysis was conducted using the randomForest package (version 4.7.1.2) with 1000 trees, mtry = 8, and Mean Decrease in Accuracy as the variable importance metric. The top 20 genes ranked by random forest were selected, and the overlapping genes identified by both methods were retained as potential biomarkers for subsequent validation [26]. Subsequently, these intersecting genes were evaluated in an independent validation cohort (GSE57148) using the Wilcoxon test to compare expression levels between disease and healthy controls [27]. Genes with a statistically significant difference (*p* < 0.05) were defined as final biomarkers. Cross-validation was conducted only in the discovery cohort (GSE47460) for model tuning and feature selection, whereas the external cohort (GSE57148) was reserved exclusively for independent validation and was not used at any stage of model training. To characterize the co-expression pattern of the five-gene diagnostic signature, pairwise Pearson correlation analyses were performed among CYP1B1, VEGFA, RET, FGG, and S100A9 in both the discovery cohort (GSE47460) and the external validation cohort (GSE57148). Considering the platform heterogeneity between the two cohorts, the analyses were conducted independently in each dataset. Correlation coefficients (r) and corresponding *p*-values were calculated based on gene expression values across samples. The correlation structure was then visualized using heatmaps, with the correlation coefficients annotated in each cell and statistical significance.

An additional exploratory subgroup analysis was performed using the dataset derived from GSE47460. Samples were categorized as control, GOLD1, GOLD2, GOLD3, and GOLD4 according to the available annotation. Expression levels of the five hub genes (CYP1B1, VEGFA, RET, FGG, and S100A9) were compared across groups and visualized as boxplots. Pairwise comparisons were performed between each GOLD subgroup and the control group using Student’s *t*-tests.

As a supplementary cross-cohort evaluation, the five-gene signature was further examined in GSE20257. After log_2_(x + 1) transformation of the expression matrix, CYP1B1, VEGFA, RET, FGG, and S100A9 were extracted and matched to the sample annotation file. A five-gene logistic regression model was then fitted within this cohort using COPD status as the binary outcome, and predicted probabilities were used to generate the combined ROC curve. ROC analysis was also performed for each individual gene. Because the combined model was fitted within GSE20257 rather than directly transferred from the discovery cohort, this analysis was treated as supplementary cohort-level support rather than strict locked external validation.

### 2.7. Comprehensive Analyses of Gene Model from SHAP Analysis to Clinical Validation

To comprehensively evaluate the five-gene diagnostic signature from both interpretability and clinical utility perspectives, two complementary analytical modules were performed: a SHAP-based interpretability module and a clinical validation module. These analyses were designed to serve different purposes. The SHAP analysis was used to improve model interpretability at the global and sample-specific levels, whereas the logistic regression and nomogram analyses were used to assess diagnostic performance and clinical applicability.

For the interpretability module, an additional xgboost classification model was constructed using the same five hub genes (CYP1B1, VEGFA, RET, FGG, and S100A9) in the discovery cohort (GSE47460) using the xgboost package (version 3.1.2.1). This auxiliary model was introduced solely to improve interpretability of the five-gene signature and did not replace the primary logistic regression-based diagnostic framework. Before model fitting, the expression values of the five genes were standardized by z-score transformation. The model was trained using COPD status as the binary outcome. After model fitting, SHAP (Shapley additive explanation) values were calculated using the shapviz package (version 0.10.3) to quantify the contribution of each feature to the predicted COPD probability. Global interpretability was assessed using a SHAP beeswarm plot, which visualized the distribution of SHAP values for each gene across all samples, and a SHAP feature-importance plot based on the mean absolute SHAP value of each feature. Local interpretability was further illustrated using representative waterfall plots for one COPD sample and one control sample, showing how individual gene contributions shifted the model prediction toward either the COPD or non-COPD class. In addition, SHAP dependence plots were generated for each of the five genes to visualize the relationship between gene expression values and their corresponding SHAP contributions, thereby illustrating gene-specific contribution patterns across the full sample set.

For clinical validation, the same five hub genes were evaluated in both the discovery cohort (GSE47460) and the external validation cohort (GSE57148) using receiver operating characteristic (ROC) analysis. The area under the curve (AUC) was calculated for each individual gene to estimate its discriminatory ability for differentiating COPD from control samples [28]. Subsequently, a multivariable logistic regression model incorporating all five genes was constructed to quantify their joint diagnostic contribution. Based on this multivariable model, a nomogram was generated to provide an individualized visual estimate of COPD probability. The diagnostic performance of the integrated model was then evaluated in the external validation cohort using ROC analysis [29]. Model calibration was assessed by comparing predicted probabilities with observed outcomes using a calibration curve, and clinical utility was evaluated by decision curve analysis (DCA) across a range of threshold probabilities. To improve interpretability of the multivariable regression model, the regression coefficients were transformed into odds ratios (ORs) with 95% confidence intervals (CIs) and visualized as a forest plot via forestplot package (version 3.2.0). Because the discovery and validation datasets were generated from different platforms, all analyses were conducted within each cohort separately, and no merged cross-platform modeling was performed.

### 2.8. Gene Set Enrichment Analysis of Each Biomarker

To further explore the biological pathways associated with each hub gene, we performed gene set enrichment analysis (GSEA) in R based on gene expression correlation. For each hub gene, Pearson correlation coefficients were calculated between its expression and that of all other genes across samples [30]. Genes were then ranked by correlation coefficients from highest to lowest, generating a ranked gene list for each hub gene. GSEA was conducted using the clusterProfiler package against the HALLMARK, REACTOME, and KEGG gene sets obtained from the MSigDB database (via msigdbr), with adjusted *p* < 0.05 considered statistically significant. The top five most significantly enriched pathways for each hub gene were visualized using ridge plots, illustrating the distribution of enrichment scores across gene sets. This analysis provided insights into the functional context of individual hub genes and their potential roles in disease pathogenesis.

### 2.9. TF–Gene Network Construction

The TF–gene network was constructed using the NetworkAnalyst tool (http://www.networkanalyst.ca/ (accessed on 4 February 2026), with transcription factor-target interactions sourced from the TRRUST database. The resulting network was then imported into Cytoscape for visualization and refinement to enhance clarity and esthetic presentation.

### 2.10. Evaluation of Immune Cell Infiltration

To assess the immune cell infiltration landscape in COPD, we applied CIBERSORT to quantify the relative proportions of 22 immune cell subtypes based on gene expression profiles. The analysis was performed in R using the CIBERSORT algorithm with the LM22 signature matrix and 100 permutations [31]. Bar plots were generated to visualize the immune cell composition in each sample, and violin plots were used to compare the infiltration levels of each immune cell type between COPD patients and healthy controls. Furthermore, to explore the potential immunomodulatory roles of the hub genes, we calculated Pearson correlation coefficients between the expression levels of hub genes and the abundance of infiltrating immune cells. The resulting correlation matrix was visualized while highlighting significant associations (*p* < 0.05). These analyses provided insights into the immune microenvironment alterations associated with COPD and the potential involvement of hub genes in immune regulation.

### 2.11. Single-Cell RNA Sequencing Data Processing and Quality Control

Single-cell RNA sequencing (scRNA-seq) data of lung tissue samples from GSE173896 were obtained, comprising 9 COPD, 7 controls. Raw data were processed using the Seurat package. Quality control excluded cells based on three metrics: number of detected genes (nFeature_RNA), UMI counts (nCount_RNA), and mitochondrial gene percentage (percent.mt). Cells with fewer than 500 genes or >15% mitochondrial reads were removed, and doublets were filtered using scDblFinder [15]. After log normalization, the top 2000 highly variable genes were selected. Data were scaled and principal component analysis (PCA) was performed. Batch effects across samples were corrected using Harmony. A shared nearest-neighbor graph was constructed on the Harmony-corrected dimensions, and cells were clustered using the Leiden algorithm at a resolution of 0.5. Clusters were visualized with UMAP and annotated based on canonical marker genes [15,32]. Differential expression between groups was identified using the FindMarkers function.

### 2.12. Bulk-Level Validation of Hub Genes in GSE173896

To further characterize the cellular localization of the hub genes, their expression patterns were examined within the GSE173896. The normalized expression values of each hub gene were projected onto the UMAP embedding using the FeaturePlot function in Seurat, allowing visualization of their distribution across cell clusters. Violin plots and dot plots were generated to compare the expression levels of hub genes among the annotated cell populations, highlighting specific enrichment.

### 2.13. Drug Prediction and Computational Docking Analysis

To identify potential therapeutic agents targeting the hub genes, drug prediction was performed using the DSigDB database via the Enrichr platform (https://maayanlab.cloud/Enrichr/ (accessed on 5 February 2026)). Concurrently, the top 150 up-regulated and down-regulated DEGs were submitted to the Connectivity Map (CMap) (https://clue.io/) for drug repurposing analysis. Candidate drugs were defined as those overlapping between DSigDB-predicted compounds and CMap-identified molecules with clear MOA and negative cs score.

For molecular docking validation, the 3D structures of selected compounds were retrieved from PubChem (https://pubchem.ncbi.nlm.nih.gov/ (accessed on 5 February 2026)) in SDF format. Energy minimization was performed in Chem3D using the MM2 force field, and the minimized structures were exported in PDB format. Protein structures of hub genes were obtained from the UniProt (https://www.uniprot.org/uniprotkb (accessed on 5 February 2026)) in PDB format. Using PyMOL(version 3.1.6.1), water molecules and organic heterogens were removed from protein structures. The prepared proteins and ligands were then imported into AutoDock Tools(version 1.5.7), where hydrogen atoms and Gasteiger charges were added, and both were saved in PDBQT format for docking. Docking grids were generated to cover the entire protein surface. Molecular docking was conducted using AutoDock Vina, and binding affinities were calculated. A binding energy < −5.0 kcal/mol was considered indicative of favorable interaction. The lowest-energy conformations were visualized using PyMOL to analyze ligand–protein interactions [33].

### 2.14. Quantitative Real-Time PCR Analysis

THP-1-derived macrophages were assigned to three groups: control, low stimulation, and high stimulation. Cells in the stimulation groups were exposed to cigarette smoke extract (CSE, Guangzhou, China; Pythonbio, Cat. No. AAPR551-2) at final concentrations of 1% and 10%, respectively, whereas untreated cells served as the control group. After treatment, total RNA was extracted from each group and reverse-transcribed into cDNA according to standard procedures. Quantitative real-time PCR (qPCR) was performed using commercially synthesized primer sets for S100A9 (Beyotime, Shanghai, China, Cat. No. QH05437S), CYP1B1 (Beyotime, Cat. No. QH02285S), VEGFA (Beyotime, Cat. No. QH06241S), FGG (Beyotime, Cat. No. QH14229S), and the internal reference gene GAPDH. Relative gene expression was calculated using the 2^−ΔΔCt^ method. Briefly, ΔCt was defined as Ct (target gene)—Ct (GAPDH), and ΔΔCt was calculated as ΔCt (sample)-mean ΔCt (control group). The control group was normalized to 1.

### 2.15. ROS Measurement

Intracellular reactive oxygen species (ROS) levels were assessed using the Reactive Oxygen Species Assay Kit with Diluent (Beyotime, Cat. No. S0034M), which is based on the DCFH-DA fluorescent probe. DCFH-DA is hydrolyzed intracellularly to non-fluorescent DCFH, which is subsequently oxidized by intracellular ROS to generate fluorescent DCF. Thus, intracellular ROS levels were quantified by measuring DCF fluorescence intensity and expressed as the median DCF fluorescence signal. In addition to the experimental groups, negative and positive control samples were included to confirm assay performance. The positive control was generated using the kit-provided Rosup reagent (50 mg/mL stock), which was used to verify the responsiveness of the ROS detection system, whereas the negative control represented the baseline fluorescence condition. For biological comparison, ROS levels were measured in the control, low stimulation, and high stimulation groups, each with three independent biological replicates.

### 2.16. Cytokine Measurement

The levels of inflammatory cytokines IL-6, IL-1β, and TNF-α in cell culture supernatants were determined using commercial ELISA kits according to the manufacturers’ instructions. The kits used were IL-6 (Merck, Darmstadt, Germany, Cat. No. RAB0307), IL-1β (Merck, Cat. No. 03-0160-00), and TNF-α (Merck, Cat. No. EZHTNFA-150K). Three independent biological replicates were analyzed for each group. Cytokine concentrations were expressed in pg/mL.

### 2.17. Statistical Analysis

**Figure 1:**
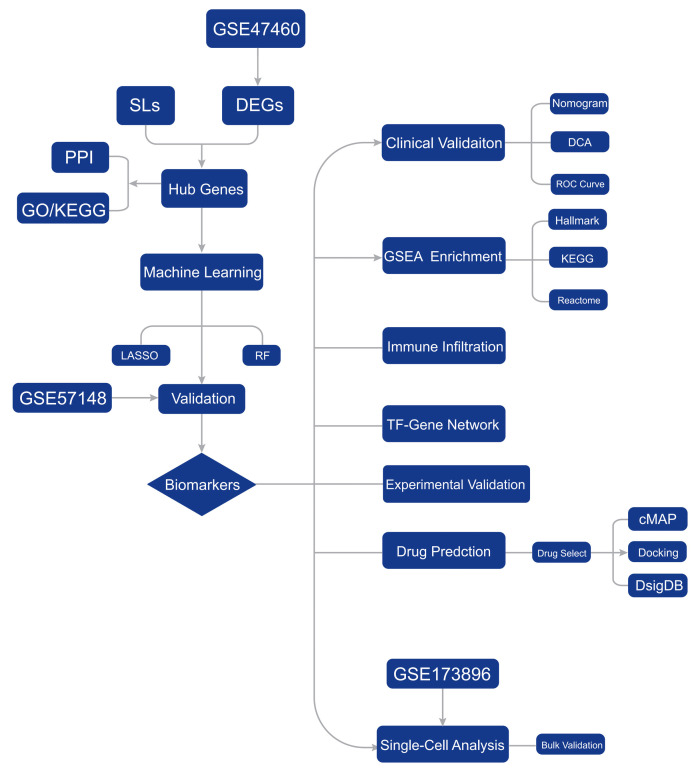
General workflow of the study.

All statistical analyses were conducted using R software (version 4.5.1). For comparisons between two groups, the Wilcoxon rank-sum test was applied. Correlations between hub gene expression and immune cell infiltration levels were assessed using Pearson correlation analysis. Receiver operating characteristic (ROC) curves were plotted and the area under the curve (AUC) was calculated to evaluate diagnostic performance using the “pROC” package. Calibration curves and the Hosmer–Lemeshow test were used to assess the goodness-of-fit of the nomogram. Decision curve analysis (DCA) was performed using the “rmda” package to evaluate clinical utility. To account for multiple testing, adjusted *p*-values were calculated using the Benjamini–Hochberg procedure where applicable. All tests were two-sided, and *p* < 0.05 was considered statistically significant. All experimental validation data were presented as mean ± SD from three independent biological replicates. For comparisons between the control group and each stimulation group, two-tailed unpaired Student’s *t*-tests were performed. Statistical significance was indicated as follows: ns, *p* ≥ 0.05; * *p* < 0.05; ** *p* < 0.01; *** *p* < 0.001.The overall workflow of this study is illustrated in Figure 1.

## 3. Results

### 3.1. Identification of Differentially Expressed Genes in COPD

**Figure 2:**
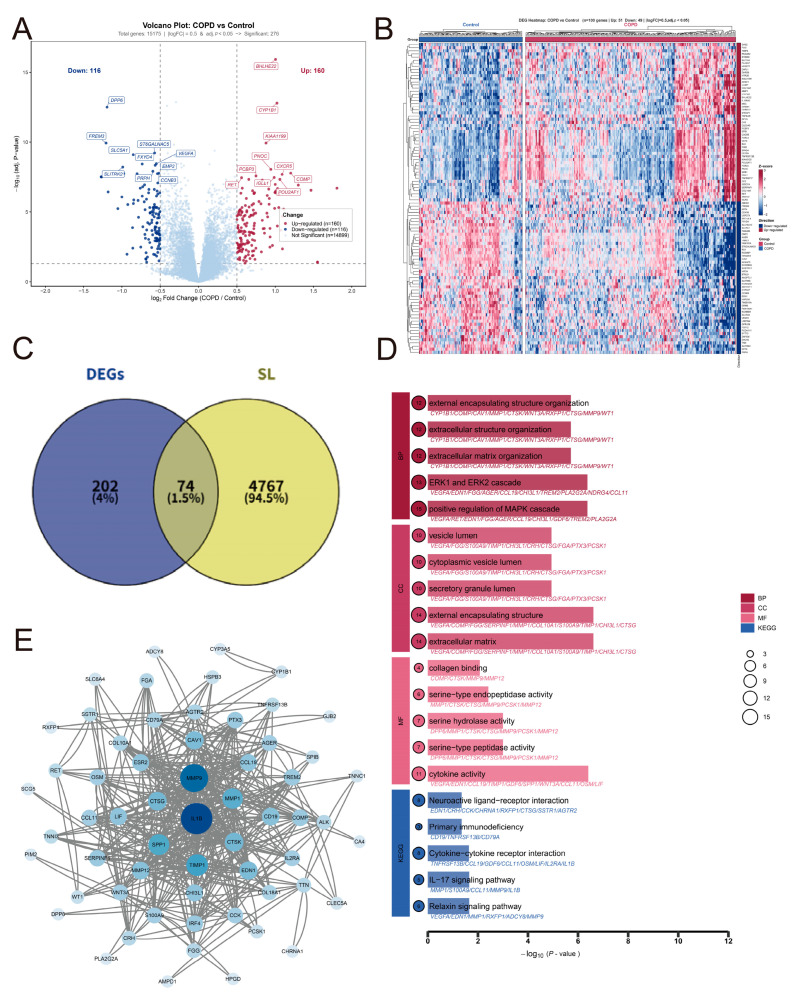
**Identification DESLGs and comprehensive analysis of DESLGs in COPD**. (**A**) Volcano plot of differentially expressed genes (DEGs) between COPD patients and healthy controls. Genes with |log_2_FC| > 0.5 and adjusted *p* < 0.05 were considered significantly differentially expressed. Specifically, significantly down-regulated genes with log_2_FC ≤ −1 are shown in purple, those with log_2_FC between −1 and −0.5 in blue; significantly up-regulated genes with log_2_FC between 0.5 and 1 in orange, and those with log_2_FC ≥ 1 in red. Gray dots represent non-significant genes. Dashed lines indicate the significance threshold (adjusted *p* = 0.05) and fold change cutoffs (|log_2_FC| = 0.5). (**B**) Heatmap of the top 100 DEGs (top 50 up-regulated and top 50 down-regulated) ranked by log_2_FC. Blue bar represents control samples, red bar represents COPD samples. Expression levels are scaled by row, with red indicating high expression and blue indicating low expression. (**C**) Venn diagram showing the intersection between DEGs and synthetic lethality-related genes (SLGs) retrieved from GeneCards (relevance score > median), yielding 74 overlapping genes defined as differentially expressed SLGs. (**D**) Protein–protein interaction (PPI) network of the 74 differentially expressed SLGs constructed using STRING (confidence score > 0.4) and visualized in Cytoscape. Node size reflects degree centrality; nodes with higher degree (e.g., IL1B, MMP9, SPP1) are highlighted as hub genes in the network. (**E**) Bar plot of Gene Ontology (GO) and Kyoto Encyclopedia of Genes and Genomes (KEGG) enrichment analysis for the 74 differentially expressed SLGs. The top five terms ranked by adjusted *p*-value are shown for biological process (BP), cellular component (CC), molecular function (MF), and KEGG pathways. Bar color represents −log_10_(*p*-value) (adjusted), and circle size indicates the number of genes enriched in each term.

Based on our selection criteria, a total of 276 DEGs were identified, comprising 160 significantly up-regulated and 116 significantly down-regulated genes. The distribution of these DEGs is visualized in a volcano plot (Figure 2A). Furthermore, hierarchical clustering of the top 100 DEGs (50 most up-regulated and 50 most down-regulated) is depicted in a heatmap, which clearly delineated the distinct expression patterns between the control group (blue) and the COPD group (red) (Figure 2B).

### 3.2. Identification of DESLGs and PPI Network Construction

We intersected the identified DEGs with a predefined list of synthetic lethality (SL) genes. This intersection yielded a total of 74 SL-related DEGs (Figure 2C) which were subsequently treated as SL-related candidate genes for downstream prioritization. A protein–protein interaction (PPI) network was constructed utilizing the STRING database with a confidence score threshold of 0.4. The network was subsequently visualized and refined using Cytoscape. Network topology analysis revealed that IL1B, MMP9, and SPP1 exhibited the highest connectivity degrees, suggesting their critical roles as hub proteins within the network (Figure 2D).

### 3.3. Functional Enrichment Analysis of DESLGs

After constructing the PPI network, Gene Ontology (GO) and KEGG pathway enrichment analyses were conducted. The top five significantly enriched terms across four categories—biological process (BP), cellular component (CC), molecular function (MF), and KEGG pathways—were visualized in a bar chart, where the circle size represents the gene count and the *x*-axis indicates the −log_10_ transformation of the adjusted *p*-values (Figure 2E).

In the BP category, the overlapping genes were predominantly enriched in the positive regulation of the MAPK cascade, the ERK1 and ERK2 cascade, and extracellular matrix (ECM) organization. For CC, the proteins were primarily localized to the extracellular matrix, external encapsulating structure, and secretory granule lumen. MF analysis highlighted significant enrichment in cytokine activity and serine-type peptidase activity. Furthermore, KEGG pathway analysis demonstrated that these SL-related DEGs were closely associated with the Relaxin signaling pathway, the IL-17 signaling pathway, and cytokine–cytokine receptor interactions.

### 3.4. Machine Learning-Based Feature Selection and Hub Gene Identification

**Figure 3:**
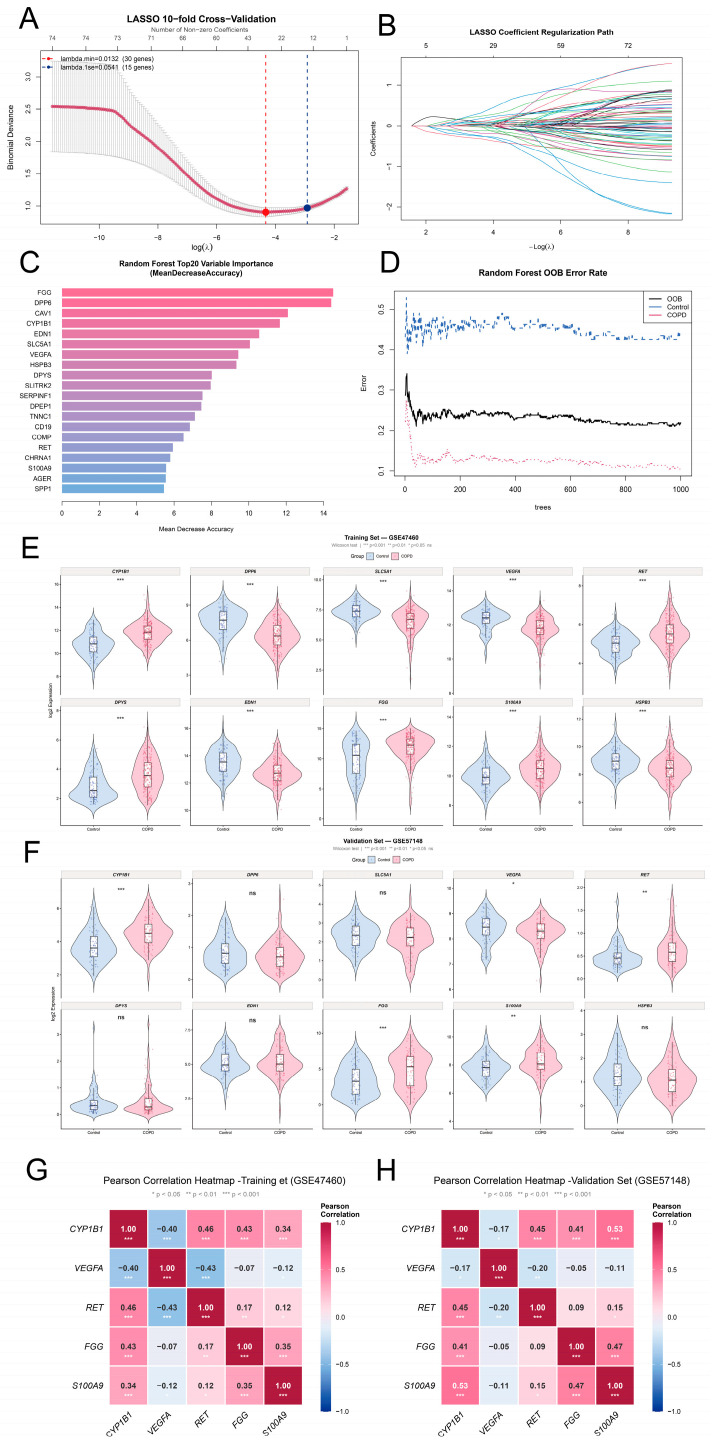
**Feature selection and validation of hub genes using machine learning algorithms.** (**A**) Cross-validation plot for tuning parameter selection in LASSO regression. The binomial deviance is plotted against log(λ). The left red dashed line indicates the optimal λ at the minimum criteria (λ.min = 0.0132), corresponding to 30 genes. The right blue dashed line indicates the λ value at one standard error (λ.1se = 0.0541), yielding 15 genes. (**B**) LASSO coefficient regularization path. Each curve represents the trajectory of a gene coefficient as the penalty parameter λ varies. Different colors indicate different variables. (**C**) Variable importance plot of the top 20 genes ranked by Mean Decrease Accuracy from random forest (RF) analysis. The top five most important genes are FGG, DPP6, CAV1, CYP1B1, and EDN1.In this plot, red indicates higher importance (higher Mean Decrease Accuracy), while blue indicates lower importance. (**D**) Out-of-bag (OOB) error rate of the random forest model. The black dashed line represents the overall OOB error, while the blue and red dashed lines indicate class-specific error rates for control and COPD groups, respectively. (**E**) Violin plots showing the expression levels of the five overlapping hub genes (CYP1B1, VEGFA, RET, FGG, S100A9) in the training cohort (GSE47460). Blue represents control samples, red represents COPD samples. (**F**) Violin plots of the same hub genes in the validation cohort (GSE57148). Consistent with the training set, VEGFA was significantly down-regulated, whereas CYP1B1, RET, FGG, and S100A9 were significantly up-regulated in COPD patients compared to controls. Statistical significance is indicated by asterisks (**G**) Pearson correlation heatmap of CYP1B1, VEGFA, RET, FGG, and S100A9 in the discovery cohort (GSE47460). (**H**) Pearson correlation heatmap of the same five genes in the external validation cohort (GSE57148). Correlation coefficients are shown in each cell. Positive correlations are shown in red and negative correlations in blue (* *p* < 0.05, ** *p* < 0.01, *** *p* < 0.001).

First, LASSO logistic regression was utilized to perform dimensionality reduction in GSE47460. Through cross-validation, the optimal penalization parameter (λ) was determined. To avoid overfitting and construct a more stringent model, the 1-standard-error criterion (λ.1se = 0.0541) was applied, which narrowed the candidates down to 15 key features (Figure 3A,B).

Concurrently, a random forest (RF) algorithm was utilized to evaluate feature importance. As the number of trees increased, the out-of-bag (OOB) error rate stabilized, demonstrating the reliability of the model (Figure 3D). Based on the Mean Decrease Accuracy metric, the top 20 most crucial genes were extracted, with FGG, DPP6, CAV1, CYP1B1, and EDN1 ranking as the top five (Figure 3C).

To enhance the reliability of the biomarker selection, the results from the LASSO (15 genes) and RF (20 genes) models were intersected, yielding 10 overlapping candidate genes. Subsequently, to ensure the reproducibility of these markers, their expression profiles were examined across the cohorts (Supplementary Figure S1). Genes exhibiting inconsistent expression trends between the discovery and validation datasets were strictly excluded.

After that, five core hub genes—CYP1B1, VEGFA, RET, FGG, and S100A9—were finalized. Violin plots confirmed their consistent and significant differential expression in both the training cohort (GSE47460, Figure 3E) and the independent validation cohort (GSE57148, Figure 3F). Specifically, VEGFA was markedly down-regulated in COPD patients, whereas CYP1B1, RET, FGG, and S100A9 were significantly up-regulated compared to the normal controls, underscoring their potential as stable diagnostic signatures.

In discovery and external validation cohorts, a LASSO-based classifier achieved AUCs of 0.887 and 0.669, respectively, indicating good training discrimination but only moderate generalizability (Supplementary Figure S2).

Analysis in GSE47460 showed CYP1B1, VEGFA, and RET altered in GOLD1 vs. controls, while FGG and S100A9 changed from GOLD2 onward. The expression of CYP1B1, RET, FGG, and S100A9 increased across GOLD stages, whereas VEGFA remained consistently lower, supporting stage-related molecular relevance of the five-gene signature (Supplementary Figure S3).

Ultimately, the Pearson correlation analysis was performed to evaluate the co-expression pattern among the five genes in the diagnostic signature (Figure 3G,H). In both GSE47460 and GSE57148, CYP1B1, RET, FGG, and S100A9 generally showed positive correlations with each other, whereas VEGFA tended to exhibit negative correlations with several of the other genes, particularly with CYP1B1 and RET. Although several gene pairs showed statistically significant correlations, the overall correlation strength was moderate rather than extreme, suggesting that the five-gene panel is not fully collinear and may provide complementary diagnostic information.

### 3.5. Integrated SHAP Interpretation and Clinical Validation Supported the Robustness of the Five-Gene Signature

**Figure 4:**
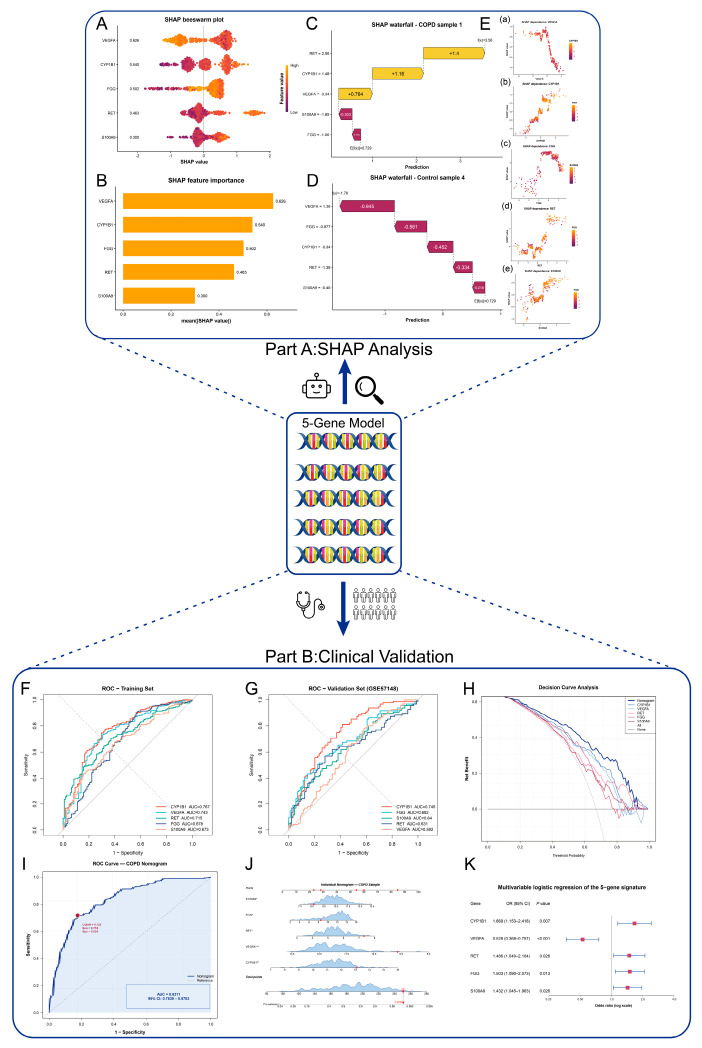
**Integrated SHAP interpretation and clinical validation of the five-gene diagnostic signature.** Part A: SHAP analysis. (**A**) SHAP beeswarm plot showing the distribution of SHAP values for the five-gene xgboost model (CYP1B1, VEGFA, RET, FGG, and S100A9). Each point represents one sample, and the *x*-axis indicates the contribution of each gene to the predicted COPD probability. (**B**) SHAP feature-importance bar plot ranking the five genes by mean absolute SHAP value. (**C**) SHAP waterfall plot for a representative COPD sample, illustrating how individual genes contribute to pushing the model prediction toward the COPD class. (**D**) SHAP waterfall plot for a representative control sample, illustrating how the overall feature contribution pattern shifts the model prediction toward the non-COPD class. (**E**) SHAP dependence plots showing the relationship between the expression values of each gene and their corresponding SHAP values. (**F**) ROC curves of the five individual genes in the discovery cohort. (**G**) ROC curves of the five individual genes in the external validation cohort (GSE57148). (**H**) Decision curve analysis (DCA) showing the net clinical benefit of the integrated model compared with individual genes and default strategies. (**I**) ROC curve of the five-gene nomogram in the validation cohort, showing the overall discriminative performance of the integrated model. (**J**) Visualization of the nomogram score distribution and a representative projected sample. *, **, *** indicate *p* < 0.05, *p* < 0.01, *p* < 0.0001, respectively; red indicates the expression profile of an example patient; the red arrow indicates the corresponding COPD probability derived from the total nomogram score. (**K**) Forest plot of the multivariable logistic regression model showing odds ratios (ORs), 95% confidence intervals (CIs), and *p*-values for the five-gene signature.The red points represent the odds ratio point estimates of each gene.

To further characterize the predictive structure of the five-gene signature, we combined SHAP-based interpretability analysis with conventional clinical validation (Figure 4). In the SHAP module, the beeswarm and feature-importance plots showed that VEGFA contributed most strongly to model output with SHAP value to 0.626, followed by CYP1B1, FGG, RET, and S100A9 from 0.3 to 0.54 (Figure 4A,B). Waterfall plots further illustrated that the direction and magnitude of feature contributions varied across samples, shifting predictions toward either the COPD or control class depending on the overall expression pattern (Figure 4C,D). Dependence plots for the five genes revealed distinct contribution profiles across the sample set, supporting the view that the panel captures partially complementary rather than redundant predictive information (Figure 4E).

In the clinical validation module, all five genes showed discriminatory capacity in the discovery cohort, with CYP1B1 and VEGFA achieving the highest AUC values of 0.767 and 0.743 (Figure 4F). Similar trends were observed in the external validation cohort, where CYP1B1 remained the best-performing single marker and FGG also retained stable predictive value (Figure 4G). The integrated model showed greater net clinical benefit across a broad range of threshold probabilities (Figure 4H), and the five-gene nomogram achieved an AUC of 0.831 (95% CI: 0.7839–0.8783) in the validation cohort (Figure 4I). The forest plot further showed that CYP1B1, RET, FGG, and S100A9 were positively associated with COPD probability with their odds’ values all above 1 while assuring their statistical significance (*p* < 0.05), whereas VEGFA showed an inverse association (Figure 4K). The calibration plot further supported the robustness of the nomogram and is shown in Supplementary Figure S4.

To further assess cross-cohort robustness, we performed a supplementary evaluation in GSE20257. In this cohort, CYP1B1 showed strong discriminatory ability (AUC = 0.877), while VEGFA (AUC = 0.729), RET (AUC = 0.677), and S100A9 (AUC = 0.670) retained moderate diagnostic value; FGG showed limited performance (AUC = 0.490). Notably, the combined five-gene model achieved an AUC of 0.907, which was higher than that of any single marker in this cohort. These findings provide additional support for the panel-level robustness of the signature, although the performance of individual genes remained heterogeneous across datasets (Supplementary Figure S5).

Taken together, these results indicate that the five-gene signature is supported by both machine learning-based interpretability and clinical predictive validation, highlighting that the panel is not only diagnostically informative but also internally explainable at both the global and individual-sample levels.

### 3.6. GSEA of Individual Hub Genes in Three Databases

**Figure 5:**
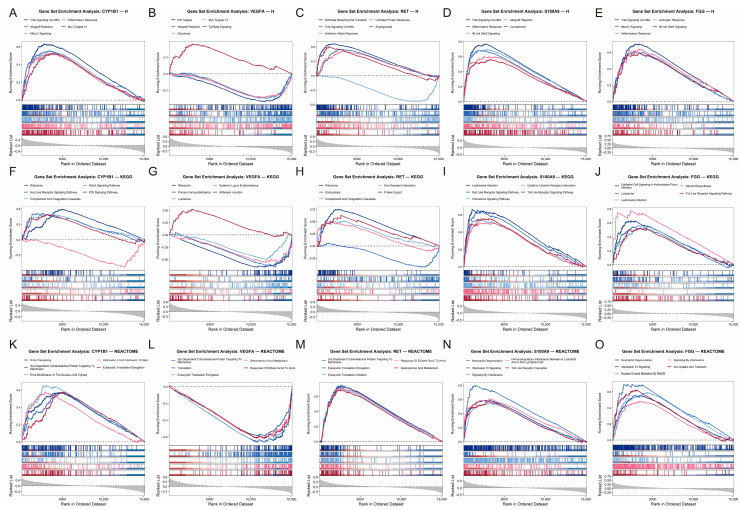
**Gene set enrichment analysis (GSEA) of individual hub genes.** For each hub gene, Pearson correlation coefficients were calculated between its expression and that of all other genes across samples. Genes were ranked accordingly and subjected to GSEA using the clusterProfiler package against the HALLMARK, KEGG, and REACTOME gene sets from MSigDB (adjusted *p* < 0.05 considered significant). The top five most significantly enriched pathways for each gene are visualized as ridge plots, showing the distribution of enrichment scores. The grey area in each ridge plot indicates the density of correlation coefficients of the hub gene with all other genes, serving as a background distribution.(**A**–**E**) Ridge plots of the top five HALLMARK pathways enriched for CYP1B1 (**A**), VEGFA (**B**), RET (**C**), S100A9 (**D**), and FGG (**E**). CYP1B1 was positively associated with TNFA signaling via NFKB, allograft rejection, MTORC1 signaling, inflammatory response, and MYC targets V1. VEGFA showed negative enrichment for E2F targets, allograft rejection, glycolysis, MYC targets V1, and positive enrichment for TGF beta signaling. RET was positively enriched for epithelial–mesenchymal transition, TNFA signaling via NFKB, unfolded protein response, angiogenesis, and negatively for interferon alpha response. S100A9 and FGG were both positively enriched for inflammatory and immune-related pathways including TNFA signaling, IL6/JAK/STAT3 signaling, and allograft rejection. (**F**–**J**) Ridge plots of the top five KEGG pathways enriched for CYP1B1 (**F**), VEGFA (**G**), RET (**H**), S100A9 (**I**), and FGG (**J**). CYP1B1 was associated with ribosome, NOD-like receptor signaling, complement cascades, p53 signaling (positive), and notch signaling (negative). VEGFA showed negative enrichment for ribosome, primary immunodeficiency, lysosome, and positive for adherens junction. RET was positively enriched for ribosome, protein export, complement cascades, ECM–receptor interaction, and negatively for endocytosis. S100A9 and FGG were both positively enriched for immune-related KEGG pathways including leishmania infection, NOD-like receptor signaling, toll-like receptor signaling, and chemokine signaling. (**K**–**O**) Ridge plots of the top five REACTOME pathways enriched for CYP1B1 (**K**), VEGFA (**L**), RET (**M**), S100A9 (**N**), and FGG (**O**). CYP1B1 was positively associated with rRNA processing, SRP-dependent co-translational protein targeting, IL-4/IL-13 signaling, and translation elongation. VEGFA showed negative enrichment for translation-related pathways including SRP-dependent targeting, eukaryotic translation elongation, and selenoamino acid metabolism. RET was positively enriched for the same translation-related pathways. S100A9 and FGG were both positively enriched for neutrophil degranulation, interleukin signaling, and innate immune pathways.

We performed correlation-based gene set enrichment analysis (GSEA) against the HALLMARK, KEGG, and REACTOME databases. The top five significantly enriched pathways for each gene were mapped using ridge plots to illustrate the distribution of enrichment scores (Figure 5A–O).

Based on the enrichment profiles, the hub genes exhibited distinct but interconnected functional trajectories. Both S100A9 and FGG demonstrated remarkably similar patterns, acting as strong positive drivers of immune and inflammatory responses. They were robustly enriched in HALLMARK pathways such as TNFA signaling via NFKB, IL6/JAK/STAT3 signaling, and general inflammatory response (Figure 5D,E). This was further corroborated by REACTOME and KEGG analyses, which highlighted their active involvement in neutrophil degranulation, toll-like/NOD-like receptor signaling cascades, and extensive interleukin signaling networks (Figure 5I,J,N,O).

Similarly, CYP1B1 was positively associated with key inflammatory cascades and MTORC1 signaling (Figure 5A). In addition to immune regulation, CYP1B1 showed significant positive enrichment in fundamental cellular activities, including ribosome biogenesis, rRNA processing, and translation elongation across the KEGG and REACTOME databases (Figure 5F,K).

RET demonstrated a distinct profile closely linked to tissue remodeling and metabolic stress. It was positively enriched for epithelial–mesenchymal transition (EMT), angiogenesis, and the unfolded protein response (Figure 5C). Additionally, RET showed strong positive associations with ECM–receptor interactions and eukaryotic translation initiation/elongation (Figure 5H,M).

In sharp contrast, VEGFA, which is the only down-regulated hub gene in our signature, has displayed widespread negative enrichment patterns. It was inversely associated with critical metabolic and proliferative pathways, including E2F targets, glycolysis, and MYC targets (Figure 5B), as well as translation-related processes and primary immunodeficiency (Figure 5G,L). Nevertheless, VEGFA exhibited positive enrichment for TGF-beta signaling and adherens junctions, underscoring its complex, bidirectional role in maintaining vascular homeostasis and structural integrity in the lung microenvironment

### 3.7. Construction of TF–Gene Regulatory Network

**Figure 6:**
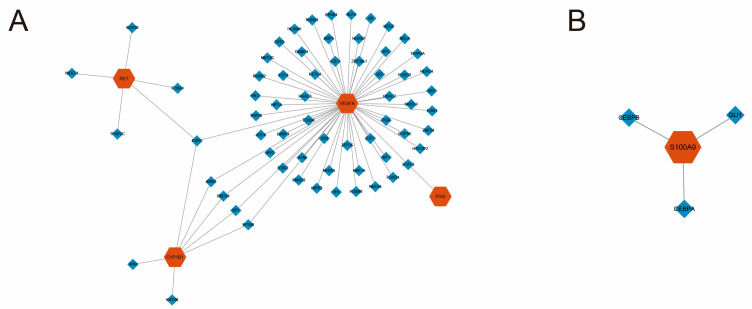
**Construction of TF–gene network.** The TF–gene interaction networks were constructed using the NetworkAnalyst tool based on the TRRUST database and visualized in Cytoscape. (**A**) TF–gene network of CYP1B1, VEGFA, RET, and FGG. Transcription factors are represented as blue diamonds, and hub genes as red hexagons. ESR1 emerged as a central regulator, connecting to RET, VEGFA, and CYP1B1. FGG was linked to VEGFA via STAT3. Additionally, ARNT, BRCA1, SP1, and EP300 were identified as common TFs regulating both VEGFA and CYP1B1, suggesting coordinated transcriptional regulation of these genes. (**B**) TF–gene network of S100A9. Unlike the other hub genes, S100A9 formed an independent regulatory network, with CEBPB, GLI1, and CEBPA as its key transcription factors, indicating distinct regulatory mechanisms underlying S100A9 expression.

To explore the upstream transcriptional regulatory mechanisms driving the expression of the five SL-related hub genes, a TF–gene interaction network was constructed using the TRRUST database and visualized via Cytoscape (Figure 6).

The network topology revealed two distinct regulatory modules. As shown in Figure 6A, four of the hub genes (CYP1B1, VEGFA, RET, and FGG) formed a highly interconnected regulatory core, suggesting a coordinated transcriptional program. Within this module, ESR1 emerged as a critical central regulator, simultaneously targeting RET, VEGFA, and CYP1B1. Furthermore, STAT3 bridged the regulation of FGG and VEGFA, while a cluster of prominent TFs, including ARNT, BRCA1, SP1, and EP300, were identified as common regulators shared by both VEGFA and CYP1B1.

Conversely, S100A9 formed an entirely independent and isolated regulatory network (Figure 6B). Its transcription was primarily driven by a distinct set of TFs, including CEBPB, GLI1, and CEBPA, indicating that the up-regulation of S100A9 in COPD is governed by separate upstream regulatory signals uncoupled from the other four hub genes.

### 3.8. Immune Cell Infiltration Landscape and Correlation with Hub Genes

**Figure 7:**
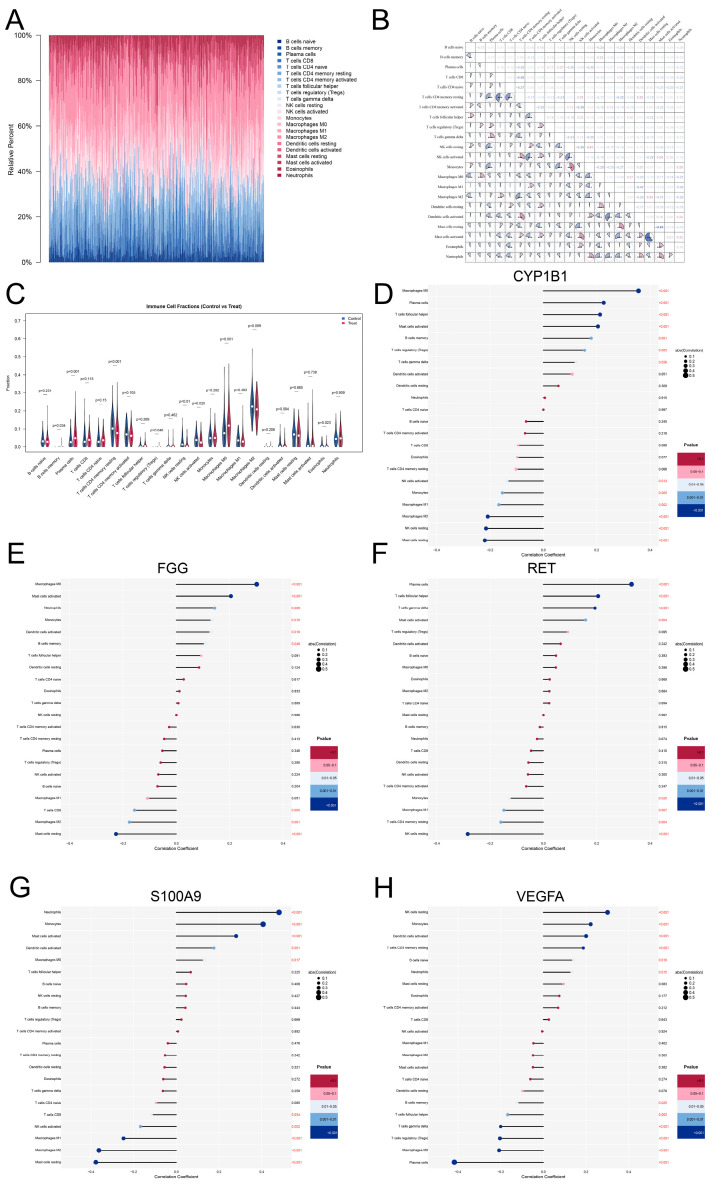
**Immune cell infiltration landscape and its correlation with hub genes in COPD.** (**A**) Bar plot showing the relative proportions of 22 immune cell subtypes in each sample, quantified by CIBERSORT. Each cell type is represented by a distinct color, and the height of the bar segment indicates its relative abundance. (**B**) Correlation heatmap of immune cell composition. The upper triangle displays Pearson correlation coefficients, with red indicating positive correlations and blue indicating negative correlations. The lower triangle represents statistical significance using a clock-style symbol: blue circles indicate negative correlations with significant *p*-values, red circles indicate positive correlations with significant *p*-values, and white (no symbol) denotes non-significant associations (*p* ≥ 0.05). Significance levels: *** *p* < 0.001, ** *p* < 0.01, * *p* < 0.05. (**C**) Violin plots comparing immune cell infiltration between COPD (red) and control (blue) samples. Plasma cells were significantly up-regulated in COPD, while T cells CD4 memory resting, NK cells resting, NK cells activated, and Eosinophils were significantly down-regulated. Macrophages M0 were significantly up-regulated, and B cells memory showed a slight but significant up-regulation with low absolute expression. *p*-values are displayed on each plot. (**D**–**H**) Lollipop plots depicting the correlation between hub gene expression and 22 immune cell types for CYP1B1 (**D**), FGG (**E**), RET (**F**), S100A9 (**G**), and VEGFA (**H**). The horizontal axis represents the Pearson correlation coefficient, with the right side indicating positive correlations and the left side negative correlations. Dot color reflects the significance level: dark blue (*p* < 0.001), blue (0.001 ≤ *p* < 0.01), light blue (0.01 ≤ *p* < 0.05), pink (0.05 ≤ *p* < 0.1), and dark red (*p* ≥ 0.1).Deeper blue represents higher significance, while deeper red represents lower significance CYP1B1 showed strong positive correlations with macrophages M0 and mast cells activated, and negative correlations with NK cells resting and mast cells resting. FGG was positively correlated with macrophages M0 and neutrophils, and negatively with mast cells resting. RET exhibited positive correlations with plasma cells and mast cells activated, and negative correlations with NK cells resting. S100A9 demonstrated strong positive correlations with neutrophils and monocytes, and negative correlations with mast cells resting and macrophages M2. VEGFA was positively correlated with NK cells resting and monocytes, and negatively correlated with plasma cells.

To characterize the immune microenvironment of COPD, the relative proportions of 22 immune cell types were quantified using the CIBERSORT algorithm (Figure 7A). Significant alterations in the immune landscape were observed between COPD and control samples (Figure 7C). Specifically, plasma cells, M0 macrophages, and memory B cells were significantly enriched in the COPD group. In contrast, resting CD4 memory T cells, both resting and activated NK cells, and Eosinophils were significantly depleted in COPD patients compared to normal controls. Furthermore, the internal correlation analysis revealed complex synergistic and antagonistic relationships within the immune microenvironment, most notably a strong inverse correlation between resting and activated mast cells (r = −0.63, *p* < 0.001) and a significant positive co-infiltration between neutrophils and monocytes (r = 0.29, *p* < 0.001) (Figure 7B).

Subsequent correlation analysis between the five-gene signature and immune cell abundance provided deeper insights into their potential roles in orchestrating the inflammatory response (Figure 7D–H). Consistent with their up-regulation in COPD, S100A9 and FGG demonstrated strong positive associations with pro-inflammatory lineages. S100A9 exhibited the strongest positive correlation with neutrophils (r = 0.48, *p* < 0.001) and monocytes (r = 0.41, *p* < 0.001), while FGG was notably associated with M0 macrophages (r = 0.30, *p* < 0.001) and neutrophils (r = 0.14, *p* < 0.01). Both genes were inversely correlated with M2 macrophages and resting mast cells, suggesting a role in promoting an active inflammatory state.

Similarly, CYP1B1 and RET were positively correlated with the cell types enriched in COPD. CYP1B1 showed the highest positive correlation with M0 macrophages (r = 0.36, *p* < 0.001) and plasma cells (r = 0.23, *p* < 0.001), while showing a significant negative link with resting NK cells. RET shared a similar profile, being positively associated with plasma cells (r = 0.33, *p* < 0.001) and activated mast cells. Conversely, VEGFA—the only down-regulated hub gene—displayed a unique and largely inverse relationship with the COPD immune landscape. It was significantly negatively correlated with plasma cells (r = −0.42, *p* < 0.001) and M0 macrophages, but showed positive associations with resting NK cells and monocytes. These findings suggest that the five-gene signature may collectively modulate the transition from immune homeostasis to chronic inflammation in the COPD lung microenvironment.

### 3.9. Single-Cell RNA Sequencing Data Quality Control and Cell Type Annotation

**Figure 8:**
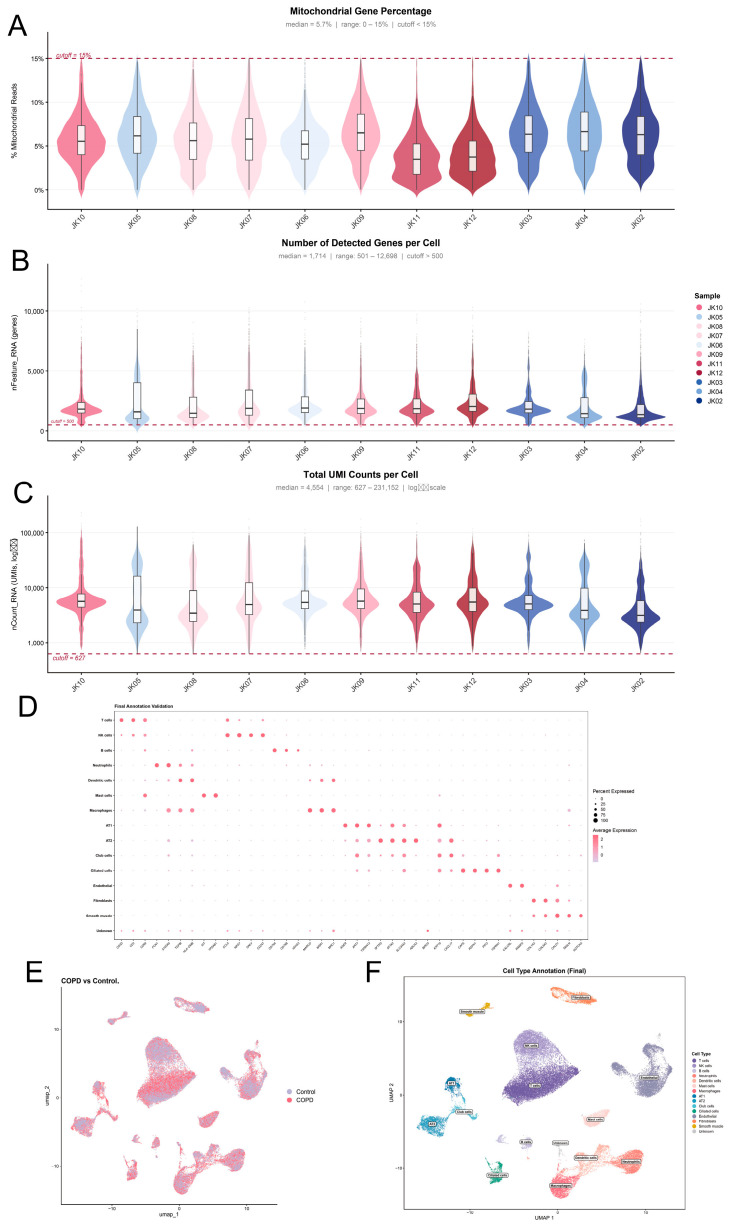
**Quality control and cell type annotation of scRNA-seq data.** (**A**) Violin plot showing the percentage of mitochondrial genes per cell across samples, with a cutoff of 15% indicated by the dashed line. Each color represents a distinct sample (labeled JK02–JK12). (**B**) Violin plot displaying the number of detected genes per cell across samples, with a cutoff of 500 genes indicated by the dashed line. (**C**) Violin plot illustrating the total UMI counts per cell across samples, with a cutoff range of 627–231,152. (**D**) Dot plot showing the expression of canonical marker genes across the 15 annotated cell types (including one unknown population). Marker genes used for cell type identification are as follows: T cells (CD3D, CD2, CD69); neutrophils (FCN1, S100A9); dendritic cells (TGFBI, HLA-DMB); mast cells (KIT, TPSAB1); NK cells (CCL5, NKG7, GNLY, CD247); B cells (CD79A, CD79B, IGHG3); macrophages (MARCO, MSR1, MRC1); alveolar type 1 cells (AGER, KRT7, TSPAN13); alveolar type 2 cells (SFTPD, SFTA2, SLC34A2, ABCA3); club epithelial cells (BIRC5, KRT19, CXCL17); ciliated epithelial cells (CAPS, RSPH1, PIFO, TSPAN1); endothelial cells (CALCRL, RAMP2); fibroblasts (COL1A2, COL6A2); smooth muscle cells (CALD1, TAGLN, NOTCH3). Dot color represents average expression level (red indicating higher expression, gray indicating lower expression), and dot size represents the percentage of cells expressing each marker gene within the cell type. (**E**) UMAP plot of all cells colored by disease status, with light purple representing control samples and red representing COPD samples. (**F**) UMAP plot of all cells colored by annotated cell types, with 15 distinct cell types visualized in different colors. Each cell cluster is labeled accordingly, demonstrating the successful identification of major cell populations in the lung tissue.

To investigate the cellular heterogeneity and the specific roles of hub genes within the COPD microenvironment at a single-cell resolution, we analyzed the scRNA-seq dataset (GSE173896). Following stringent QC procedures, a total of 46,850 high-quality single cells were retained from 11 lung tissue samples (labeled JK02–JK12). These consisted of 25,184 cells from six COPD patients and 21,666 cells from five control subjects (comprising three never-smokers and two non-COPD smokers). The QC metrics demonstrated robust data viability: cells were filtered to ensure the proportion of mitochondrial genes was below 15% (Figure 8A), the number of detected genes per cell exceeded 500 (Figure 8B), and total unique molecular identifier (UMI) counts ranged appropriately from 627 to 231,152 (Figure 8C).

Following data normalization and principal component analysis (PCA), Uniform Manifold Approximation and Projection (UMAP) was utilized for non-linear dimensionality reduction and visualization. As shown in Figure 8E, the cells were successfully segregated by disease status (COPD vs. control).

Subsequently, unsupervised clustering identified 15 distinct cell clusters, which were robustly annotated into 14 known cell lineages and one unknown population based on the expression profiles of canonical marker genes (Figure 8D,F). Specifically, the immune compartment was identified utilizing the following markers: T cells (CD3D, CD2, CD69), NK cells (CCL5, NKG7, GNLY, CD247), B cells (CD79A, CD79B, IGHG3), macrophages (MARCO, MSR1, MRC1), neutrophils (FCN1, S100A9), dendritic cells (TGFBI, HLA-DMB), and mast cells (KIT, TPSAB1).

Concurrently, the structural and epithelial compartments were clearly delineated: alveolar type 1 (AT1) cells were marked by AGER, KRT7, and TSPAN13, while alveolar type 2 (AT2) cells were characterized by SFTPD, SFTA2, SLC34A2, and ABCA3. Other respiratory epithelial lineages included club cells (BIRC5, KRT19, CXCL17) and ciliated epithelial cells (CAPS, RSPH1, PIFO, TSPAN1). Furthermore, mesenchymal and vascular populations were mapped, including endothelial cells (CALCRL, RAMP2), fibroblasts (COL1A2, COL6A2), and smooth muscle cells (CALD1, TAGLN, NOTCH3). The distinct expression patterns of these markers across clusters confirmed the reliability of our cell type annotation (Figure 8D), laying the foundation for downstream spatial and cell-specific transcriptomic analyses.

### 3.10. Single-Cell Expression Landscape of Hub Genes in COPD

**Figure 9:**
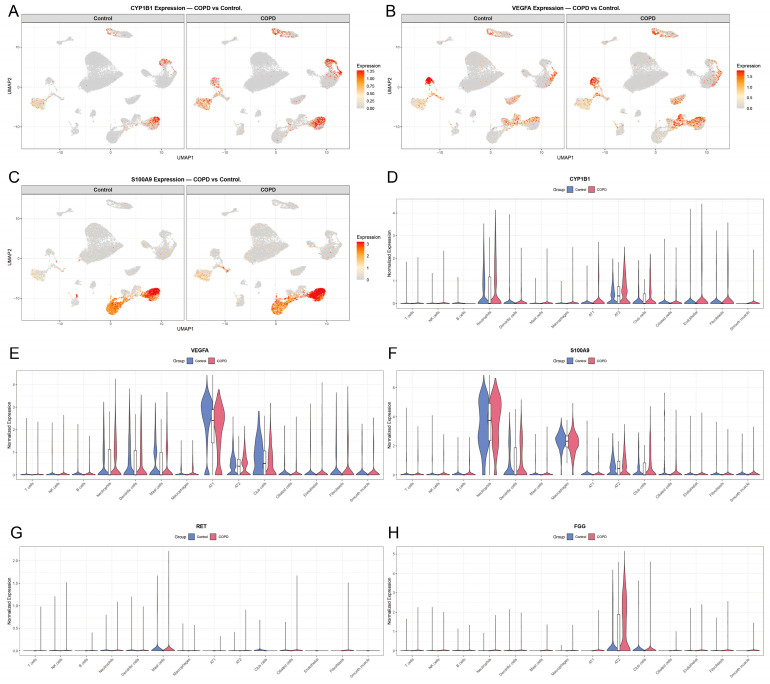
**Single-cell expression landscape of hub genes in COPD and control lung tissues**. (**A**–**C**) UMAP feature plots showing the expression distribution of CYP1B1 (**A**), VEGFA (**B**), and S100A9 (**C**) across all cells. Red indicates high expression, yellow indicates moderate expression, and gray indicates low or no expression. (**D**–**H**) Violin plots depicting the expression levels of CYP1B1 (**D**), VEGFA (**E**), S100A9 (**F**), RET (**G**), and FGG (**H**) across 14 annotated cell types in COPD (red) and control (blue) samples. CYP1B1 was broadly expressed across immune and structural compartments including neutrophils, AT2 cells and club cells. In COPD, it was significantly up-regulated in neutrophils, AT1 cells, AT2 cells, club cells, ciliated cells, endothelial cells, fibroblasts, and smooth muscle cells. Although mast cells and macrophages also showed increased expression, their overall expression levels remained low. VEGFA was primarily expressed in AT1 cells, club cells, neutrophils, dentritic cells, mast cells and AT2 cells. In COPD, it was significantly up-regulated in neutrophils and AT2 cells but markedly down-regulated in AT1 cells, mast cells and club cells. S100A9 was predominantly expressed in neutrophils, dendritic cells, AT2 cells, club cells and macrophages, with substantial up-regulation in COPD while being down-regulated in neutrophils slightly. It was also significantly increased in T cells, B cells, and ciliated cells though these populations are low. RET expression was largely restricted to mast cells and club cells, with a slight but significant down-regulation of mast cells in COPD. FGG showed the highest expression in AT2 cells, where it was significantly up-regulated in COPD. Modest increases were observed in T cells, NK cells, neutrophils, dendritic cells, mast cells, and macrophages, but these populations exhibited low overall expression levels.

To elucidate the cellular origins and spatial distribution of the five hub genes, their expression profiles were projected onto the UMAP coordinate space and compared across 15 annotated cell types between COPD and control samples (Figure 9). UMAP feature plots showed the distribution patterns of CYP1B1, VEGFA, and S100A9 (Figure 9A–C). Violin plots revealed cell type-specific expression differences (Figure 9D–H). Detailed information is collected in Supplementary Table S2.

Violin plots were utilized to quantitatively assess expression alterations across individual cell types (Figure 9D–H). CYP1B1 was broadly detected in both immune and structural compartments, with notable basal expression in neutrophils, AT2 cells, and club cells. In the COPD cohort, CYP1B1 was significantly up-regulated across multiple lineages, including neutrophils, AT1 cells, AT2 cells, club cells, ciliated cells, endothelial cells, fibroblasts, and smooth muscle cells. Furthermore, significant increases were observed in mast cells and macrophages, although their absolute expression levels remained relatively low.

The expression of VEGFA was primarily localized to AT1 cells, club cells, neutrophils, dendritic cells, mast cells, and AT2 cells. Differential analysis revealed a heterogeneous regulation pattern in COPD. VEGFA was significantly up-regulated in neutrophils and AT2 cells, whereas it demonstrated marked down-regulation in AT1 cells, mast cells, and club cells.

S100A9 expression was predominantly concentrated in neutrophils, dendritic cells, AT2 cells, club cells, and macrophages. In COPD patients, S100A9 was substantially up-regulated across the majority of these populations, with the exception of neutrophils, which exhibited a slight down-regulation. Additionally, significant up-regulation was observed in T cells, B cells, and ciliated cells, despite low overall expression abundance in these groups.

RET displayed the most restricted expression pattern among the hub genes, being largely confined to mast cells and club cells. In the COPD context, a slight but statistically significant down-regulation of RET was exclusively observed in mast cells.

Finally, FGG exhibited its highest basal expression within the AT2 cell population, where it was significantly up-regulated in COPD samples. While modest but significant increases were also detected in T cells, NK cells, neutrophils, dendritic cells, mast cells, and macrophages, the absolute expression of FGG within these immune populations remained low.

### 3.11. Candidate Drug Prediction and Computational Docking Analysis

To identify potential therapeutic compounds targeting the identified hub genes, we integrated results from the Connectivity Map (CMap) and the DSigDB database via the Enrichr platform. A total of 178 candidate drugs were identified (Supplementary Table S3). Based on their pharmacological mechanisms and relevance to COPD pathogenesis, three representative compounds—Rutin, Methazolamide, and Miglitol—were selected for further investigation (Table 2).

Molecular docking was performed to evaluate the binding affinity between these compounds and their respective hub gene targets. Notably, since VEGFA was significantly down-regulated in COPD, we conducted molecular docking for Rutin against its downstream receptor, ADORA2A, to assess its potential in modulating the VEGFA-related signaling axis, alongside its direct target CYP1B1.

**Figure 10:**
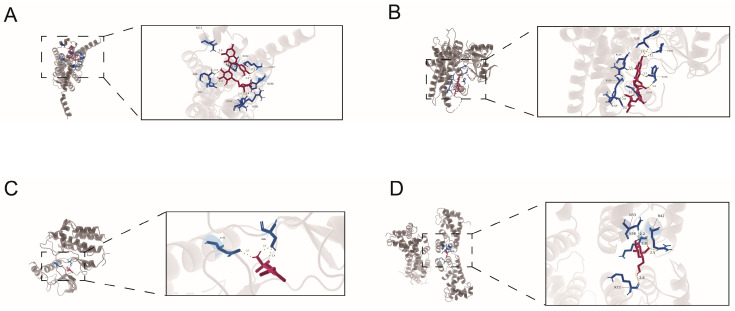
**Computational docking analysis of candidate compounds with hub gene-related targets.** (**A**) Molecular docking of Rutin with ADORA2A (receptor of VEGFA). The protein receptor is shown in gray, the docking protein in blue, and the drug ligand (Rutin) in red. Yellow dashed lines indicate hydrogen bonds, with bond lengths labeled. (**B**) Molecular docking of Rutin with CYP1B1. Rutin exhibited stable binding interactions with CYP1B1, with multiple hydrogen bonds forming between the ligand and key residues. (**C**) Molecular docking of Methazolamide with RET. Methazolamide formed hydrogen bonds with RET, supporting its prioritization as a candidate compound for future testing. (**D**) Molecular docking of Miglitol with S100A9. Miglitol demonstrated favorable binding affinity with S100A9, with hydrogen bonds contributing to the interaction stability.

The docking analysis suggested favorable predicted binding for all tested pairs. Rutin exhibited the highest binding affinity with both ADORA2A (−8.4 kcal/mol) and CYP1B1 (−8.2 kcal/mol) (Figure 10A,B). Multiple hydrogen bonds were formed between Rutin and key amino acid residues of the receptors, ensuring a stable complex. Methazolamide showed acceptable binding capability with RET, yielding a binding energy of −5.8 kcal/mol (Figure 10C). Similarly, Miglitol demonstrated favorable binding stability with S100A9, with an acceptable binding energy of −5.1 kcal/mol supported by a network of hydrogen bonds (Figure 10D). These findings support these compounds as preliminary candidates for further validation, but do not establish therapeutic efficacy or target specificity in COPD.

### 3.12. ROS Accumulation Was Markedly Increased After Stimulation

**Figure 11:**
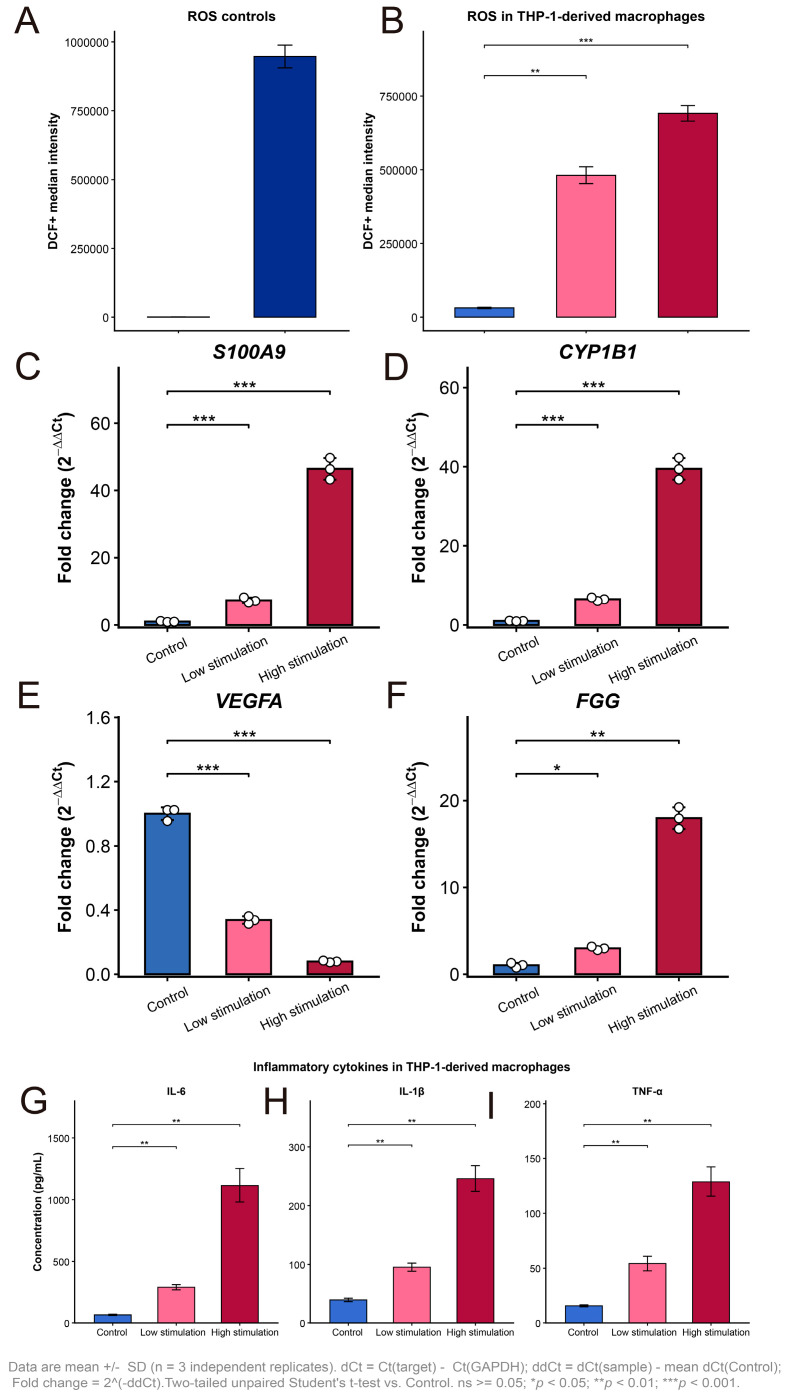
Experimental validation of oxidative stress, gene expression changes, and inflammatory activation in THP-1-derived macrophages. (**A**) ROS assay quality controls showing the expected separation between the negative and positive controls. (**B**) ROS levels in THP-1-derived macrophages under control, low stimulation, and high stimulation conditions. ROS intensity was measured as median DCF fluorescence. Both stimulation groups showed significantly increased ROS levels compared with the control group. (**C**–**F**) qPCR validation of the four candidate genes S100A9 (**C**), CYP1B1 (**D**), VEGFA (**E**), and FGG (**F**). Relative expression was calculated using the 2^−ΔΔCt^ method with GAPDH as the internal control and normalized to the control group. S100A9, CYP1B1, and FGG were up-regulated after stimulation, whereas VEGFA was down-regulated. (**G**–**I**) ELISA quantification of inflammatory cytokines IL-6 (**G**), IL-1β (**H**), and TNF-α (**I**) in cell culture supernatants. Cytokine levels were significantly elevated in the stimulated groups, with the highest levels observed in the high stimulation group. Data are presented as mean ± SD from three independent biological replicates. Statistical comparisons were performed using two-tailed unpaired Student’s *t*-tests versus the control group. ns, *p* ≥ 0.05; * *p* < 0.05; ** *p* < 0.01; *** *p* < 0.001.

To evaluate oxidative stress in THP-1-derived macrophages, intracellular ROS levels were measured by DCF fluorescence. As shown in Figure 11A, the negative and positive controls showed the expected separation, confirming the reliability of the assay. Compared with the control group, ROS levels were markedly elevated in both the low stimulation and high stimulation groups (Figure 11B). The increase was already significant under low stimulation and became more pronounced under high stimulation, indicating a dose-dependent enhancement of oxidative stress following stimulation.

### 3.13. qPCR Validation Confirmed Stimulation-Responsive Dysregulation of COPD-Related Genes

We next examined the expression of four candidate genes by qPCR. Given that RET showed the most restricted cell type distribution in the single-cell analysis, being largely confined to mast cells and club cells, it was not included in the current THP-1-derived macrophage validation experiments. Instead, S100A9, CYP1B1, VEGFA, and FGG were selected for validation because they were more closely related to inflammatory or stress-responsive programs and were considered more suitable for this macrophage-based model. Consistent with the transcriptomic findings, S100A9 and CYP1B1 were strongly up-regulated after stimulation, with both genes showing a stepwise increase from the low stimulation group to the high stimulation group (Figure 11C,D). FGG was also significantly increased, although the magnitude of induction was lower under low stimulation than that observed for S100A9 and CYP1B1 (Figure 11F). In contrast, VEGFA was down-regulated following stimulation, and this decrease became more evident in the high stimulation group (Figure 11E). Overall, these results support that stimulation induced a gene expression pattern characterized by increased inflammatory- and stress-related markers together with reduced VEGFA expression.

### 3.14. Inflammatory Cytokines Were Significantly Elevated in Stimulated Macrophages

To further assess inflammatory activation, the secretion of IL-6, IL-1β, and TNF-α was measured by ELISA. As shown in Figure 11G–I, all three cytokines were significantly increased in the low stimulation group compared with the control group and were further elevated in the high stimulation group. Among them, IL-6 showed the most marked increase, followed by IL-1β and TNF-α. These findings indicate that stimulation induced a robust inflammatory phenotype in THP-1-derived macrophages.

## 4. Discussion

Chronic obstructive pulmonary disease remains a heterogeneous and slowly progressive disorder in which persistent inflammation, epithelial injury, extracellular matrix remodeling, and vascular dysfunction converge to drive irreversible airflow limitation. Beyond differential expression, a practical goal is to prioritize biologically plausible vulnerabilities that may warrant future perturbation-based testing [34]. Synthetic lethality provides a useful conceptual framework for this goal because it captures context-specific vulnerabilities where a cell state tolerates a single perturbation but becomes nonviable when a second buffering pathway is inhibited [35]. Recent advances in curated resources and computational frameworks have broadened access to synthetic lethality knowledge and have made it feasible to integrate this information with disease omics for target discovery [36].

In this study, a COPD-associated transcriptomic signature was first established in bulk lung tissue, yielding 276 differentially expressed genes with clear separation between COPD and control. Interacting these genes with a curated synthetic lethality gene set narrowed the search space to 74 synthetic lethality-related differentially expressed genes. This filtering step is not intended to claim validated lethal gene pairs in COPD tissue. Instead, it prioritizes genes that have been repeatedly implicated as components of synthetic lethal relationships across experimental and curated evidence, thereby enriching for nodes that may represent conditional essentiality under disease stress [37].

The protein interaction topology and functional enrichment of these synthetic lethality-related differentially expressed genes pointed to a coherent biological theme. Enrichment in MAPK and ERK cascades, extracellular matrix organization, cytokine activity, and IL 17-related pathways is consistent with the inflammatory and remodeling dominated COPD microenvironment [38,39]. The PPI network highlighted IL1B, MMP9, and SPP1 as highly connected nodes, suggesting that synthetic lethality-related genes are embedded in the same inflammatory proteolytic circuits that shape tissue damage and repair [40,41]. While hubness does not prove causality, it supports the idea that COPD-associated transcriptional programs converge on a limited set of network bottlenecks that may become targetable vulnerabilities when buffering capacity is exhausted.

A key contribution of the present work is the transition from network-level candidates to a reproducible and clinically oriented gene panel. By combining LASSO and random forest feature selection and enforcing consistency across an independent validation cohort, five core hub genes were finalized, namely CYP1B1, VEGFA, RET, FGG, and S100A9. The resulting nomogram achieved strong discrimination in the validation cohort, exceeding the performance of individual genes. This result supports a multi-gene strategy that better reflects COPD biology, where multiple parallel processes jointly determine disease status and severity.

Biological interpretation of the five-gene signature aligns with known COPD mechanisms and strengthens plausibility. S100A9 is a prototypic inflammatory mediator linked to cigarette smoke-induced lung injury and functional decline in experimental COPD models, providing mechanistic support for its up-regulation and for its strong correlation with neutrophil and monocyte abundance observed here [42]. FGG has been reported as up-regulated in COPD lung tissue and associated with immune-related pathway enrichment, supporting its role as a biomarker candidate and its linkage to macrophage-rich inflammatory states [43]. CYP1B1 emerged as the strongest single gene discriminator and showed broad single-cell expression across immune and structural compartments. A recent mechanistic study demonstrated that oxidative stress induces CYP1B1 and promotes lipid-laden macrophage formation in COPD-relevant settings, which is consistent with our observation of positive associations with macrophage-related infiltration signals [44].

VEGFA was the only down-regulated gene in the bulk derived signature, yet its regulation appeared cell type dependent in single-cell analysis, with down-regulation in specific epithelial and stromal populations accompanied by up-regulation in selected immune or progenitor-like contexts. This heterogeneity may reconcile prior evidence that chronic smoke exposure can deplete protective VEGF-mediated barrier functions and permit-amplified inflammatory cascades [45]. The relevance of VEGF signaling to alveolar and microvascular integrity is also supported by work showing that epithelial VEGFA is a key determinant of specialized endothelial populations in the lung, highlighting why reduced VEGF activity could plausibly contribute to structural vulnerability in COPD [46]. RET showed the most restricted cellular distribution, primarily in mast cells and club cells in our dataset, suggesting that its contribution may be confined to discrete epithelial-immune niches rather than representing a ubiquitous COPD marker. Because direct mechanistic evidence connecting RET to COPD pathogenesis remains limited compared with the other four genes, it should be viewed as a high-priority hypothesis requiring targeted validation in relevant cell types.

The immune deconvolution analysis revealed a COPD-associated shift toward plasma cells, macrophage subsets, and reduced resting lymphoid populations, and the hub genes displayed coherent correlations with this landscape. CIBERSORTx-based digital cytometry has been widely used to infer immune composition from bulk transcriptomes and provides a pragmatic bridge between bulk discovery cohorts and immune hypotheses [47]. The strong association of S100A9 with neutrophils and monocytes aligns with reports of systemic and functional neutrophil alterations in early stage COPD, supporting the clinical relevance of neutrophil-linked transcriptional programs [48]. The divergence of VEGFA correlations from the pro-inflammatory genes suggests that the five-gene panel jointly captures both inflammatory amplification and the loss of tissue-protective programs, a duality also reflected by VEGF deficiency-driven IL 33-associated inflammatory escalation in a murine COPD model.

Single-cell analysis added an essential layer of mechanistic resolution by mapping hub gene expression to specific cell lineages and by validating that the bulk signature is not driven by a single dominant population. The identification of multiple epithelial and stromal compartments with altered CYP1B1 expression and the presence of inflammatory-associated epithelial subsets in COPD are consistent with prior single-cell studies describing epithelial heterogeneity and ectopic inflammatory programs in COPD lungs [49]. This cell type localization is particularly important for synthetic lethality-motivated interpretation, because synthetic lethal dependencies are often highly context-specific and may only manifest in selected cell states such as stressed epithelium, activated macrophages, or remodeling-associated stromal cells.

From a synthetic lethality perspective, the present results suggest a practical roadmap for COPD precision targeting. The synthetic lethality-related filtering step prioritized genes with evidence of conditional essentiality in other biological contexts, then multi-cohort validation refined them into a reproducible diagnostic panel. The next translational step is to convert this gene list into COPD-relevant synthetic lethal hypotheses by identifying partner genes and pathways that are uniquely required in hub gene high COPD-like cell states. This can be addressed experimentally through combinatorial perturbation screening in primary airway epithelial cultures, organoids, or macrophage models under cigarette smoke and inflammatory cytokine stress, followed by validation in ex vivo lung tissue. Such a strategy would directly test whether COPD-associated transcriptional rewiring creates exploitable buffering dependencies, which is the defining promise of synthetic lethality [46].

Drug prediction and docking analyses provided an initial therapeutic hypothesis layer. While the predicted compounds and docking scores suggest feasible binding interactions, these results remain computational and should be interpreted as prioritization rather than proof of efficacy. The most informative follow-up would pair compound testing with functional readouts in hub gene high-cell populations, combined with perturbation experiments to confirm that observed effects depend on the intended targets and reflect a synthetic lethality-like vulnerability rather than nonspecific anti-inflammatory activity [50].

Compared with previously reported COPD diagnostic models derived primarily from conventional differential expression screening followed by machine learning, the present study adds an SL-related prioritization step before feature selection, thereby narrowing the candidate space toward genes linked to stress-response and context-dependent vulnerability biology. In addition, the identified five-gene panel was examined not merely in bulk transcriptomic cohorts but in single-cell and immune infiltration analyses as well, which provided additional biological context for the selected markers. Importantly, these preliminary experiments support the biological relevance of the identified genes under inflammatory stress. Nevertheless, our findings should be interpreted in the context of public dataset-based validation. Although the included cohorts were derived from populations in the United States, South Korea, and Japan, the present study was not specifically designed to establish ethnic generalizability, and further validation in well-annotated multi-center cohorts from broader populations is still required.

Accordingly, the five-gene panel should be interpreted as a supplementary molecular aid for COPD diagnosis and risk stratification rather than a replacement for conventional spirometry-based assessment. The additional analysis in GSE20257 further supports the robustness of the integrated five-gene panel at the cohort level, as the combined model achieved an AUC of 0.907. However, the performance of individual genes was not fully uniform across datasets, with FGG showing limited discriminatory value in this supplementary cohort. This pattern suggests that the clinical relevance of the signature is better interpreted at the integrated panel level rather than through any single gene alone. At the same time, because the combined model in GSE20257 was fitted within that dataset, these findings should be interpreted as supportive supplementary evidence rather than definitive prospective clinical validation.

Several limitations should be considered. The synthetic lethality gene set is largely derived from cancer and perturbation screens performed in proliferative systems, so COPD-specific genetic interaction structure may differ [51]. Bulk transcriptomes are sensitive to changes in cellular composition, and although digital cytometry and single-cell validation mitigate this concern, causal directionality cannot be inferred [52]. In addition, incomplete FEV1/FVC data across the public cohorts precluded standardized GOLD-stage stratification and limited severity-based subgroup analyses. The single-cell cohort size and clinical heterogeneity may limit generalizability of cell type-specific conclusions. It should also be noted that the different algorithms used in this study were not intended as fully interchangeable diagnostic models. Rather, they were applied in a complementary manner: LASSO and random forest for robust feature prioritization, logistic regression for clinically interpretable prediction, and xgboost for post hoc SHAP-based explanation. This design was chosen to balance model stability, interpretability, and translational readability. Finally, although preliminary validation in THP-1-derived macrophages supported stimulation-associated ROS accumulation, the dysregulation of S100A9, CYP1B1, VEGFA, and FGG, and increased inflammatory cytokine production, these experiments were limited to a single in vitro macrophage model and do not yet establish cell type specificity, causal function, or COPD-specific synthetic lethal interactions.

In summary, this study reframes COPD biomarker discovery through a synthetic lethality-inspired lens by integrating differential expression, network analysis, machine learning validation, immune deconvolution, and single-cell mapping. The resulting five-gene signature provides a robust diagnostic model and generates mechanistically grounded hypotheses about inflammation-driven vulnerabilities and the loss of protective vascular epithelial programs. Defining COPD-relevant synthetic lethality-like interactions involving these hub genes represents a logical next step toward functional target discovery and mechanistic validation. Future work should extend validation to epithelial and mast cell-relevant systems, particularly for RET, to better match the cell type context suggested by the single-cell data.

## 5. Conclusions

In conclusion, we identified and validated a five-gene diagnostic signature for COPD comprising CYP1B1, VEGFA, RET, FGG, and S100A9. The integrated panel showed reproducible diagnostic performance across independent cohorts and was further supported by enrichment analysis, immune deconvolution, single-cell mapping, and preliminary in vitro experiments. Together, these findings support SL-related gene prioritization as a useful hypothesis-generating framework for COPD biomarker discovery and for the development of an adjunctive molecular diagnostic aid. Further studies are needed to validate the signature in broader clinical cohorts and additional cell types, clarify the cell context-dependent role of RET, and determine whether these genes participate in true COPD-relevant synthetic lethality-like interactions.

## Figures and Tables

**Table 1:** Basic information of datasets used in this study.

Dataset	Platform	Sample Size	Age	Sex	Smoking Status		FEV1%		Sample Source	Authors	Country	Last Update
		**(COPD/Controls)**			COPD	Control	COPD	Control				
GSE47460	GPL6480	220/108	62.63 ± 10.41	55 male	3 current 66 former 3 never	15 former 8 never	44.6 ± 24.1	78.8 ± 28.7	Lung	Tedrow et al.	USA	1 October 2019
				37 female								
	GPL14550											
GSE57148	GPL11154	98/91	67.5 ± 6.4	189 male	Unknown		71.9 ± 13.4	91.0 ± 12.4	Lung	Kim WJ et al.	South Korea	15 May 2019
GSE47460	GPL570	23/112	43.69 ± 9.88	95 male 40 female	All current	53 current 59 never	Unknown		Lung	Shaykhiev et al.	USA	25 March 2019
GSE173896	GPL20795	9/7	64.2 ± 13.2	Unknown	Unknown		84.1 ± 8.7	96.4 ± 10.0	Lung	Watanabe N et al.	Japan	15 November 2023

**Table 2:** Drugs used for docking.

Drug Name	Norm cs	MOA	Genes
Rutin	−1.2864	Antioxidant|Capillary stabilizing agent|Nitric oxide scavenger	CYP1B1; VEGFA
Methazolamide	−1.1488	Carbonic anhydrase inhibitor	RET
Miglitol	−0.872	Glucosidase inhibitor	S100A9

## Data Availability

The datasets analyzed in this study are publicly available in the Gene Expression Omnibus (GEO) repository (https://www.ncbi.nlm.nih.gov/geo/ (accessed on 3 February 2026)) under the accession numbers GSE47460, GSE57148, and GSE173896.

## Supplementary Materials

The following supporting information can be downloaded at: https://www.mdpi.com/article/10.3390/cimb48050475/s1.
