# Supplementary material for: A Synthetic Lethality-Informed Multi-Omic Framework for Identifying a Five-Gene Diagnostic Signature in Chronic Obstructive Pulmonary Disease
Source: Curr Issues Mol Biol. 2026 May 2;48(5):475. doi: 10.3390/cimb48050475 (PMC13204272; doi:10.3390/cimb48050475)
Supplement: Supplementary file 1 [file cimb-48-00475-s001.zip › Supplementary Figures caption & Tables caption.pdf]

supplementary materials

Supplementary Figure S1: Venn diagram showing the overlap between genes selected by LASSO regression (15 genes) and random forest (RF) model (20 genes). The intersection yielded 10 common candidate genes, which were further evaluated for reproducibility across cohorts.

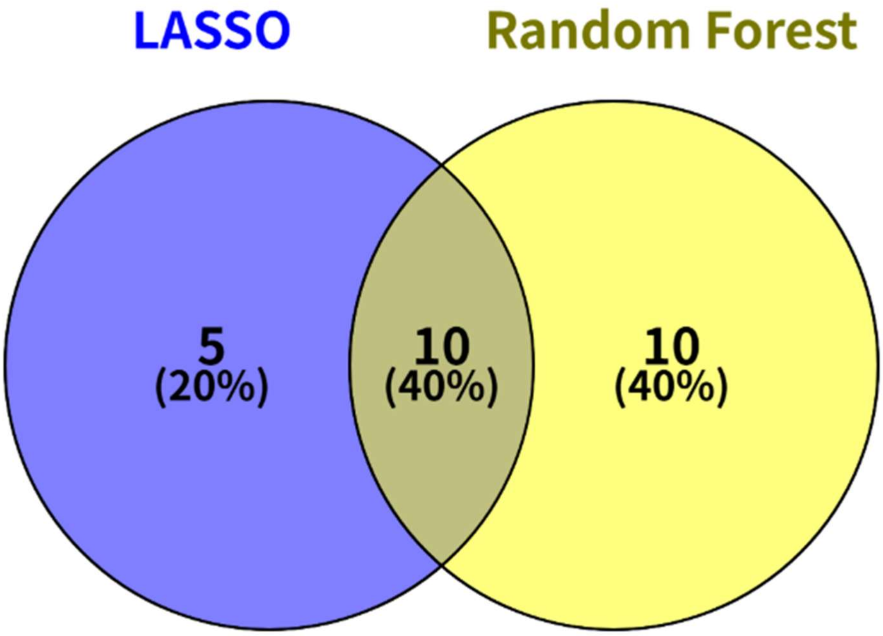

Supplementary Figure S2. Receiver operating characteristic (ROC) curves of the LASSO-based classifier in the discovery cohort (blue, AUC = 0.887) and the external validation cohort (red, AUC = 0.669). The model demonstrated good discrimination in the training set but only moderate generalizability in the validation set.

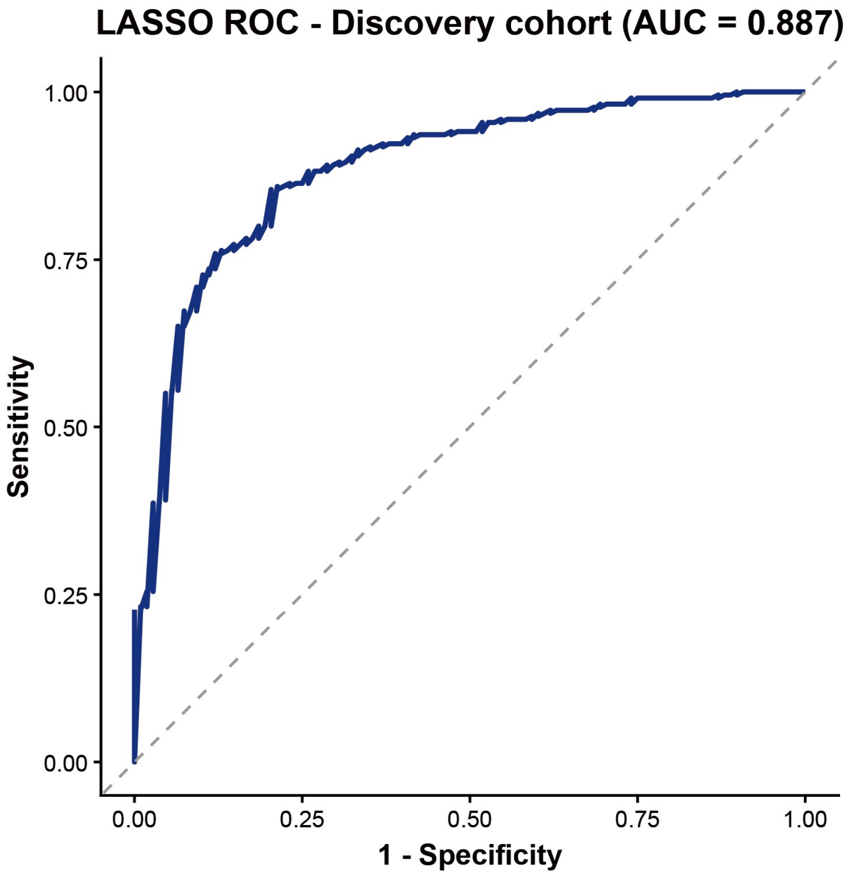
LASSO ROC - Discovery cohort (AUC = 0.887)
Sensitivity
1 - Specificity
0.00
0.25
0.50
0.75
1.00

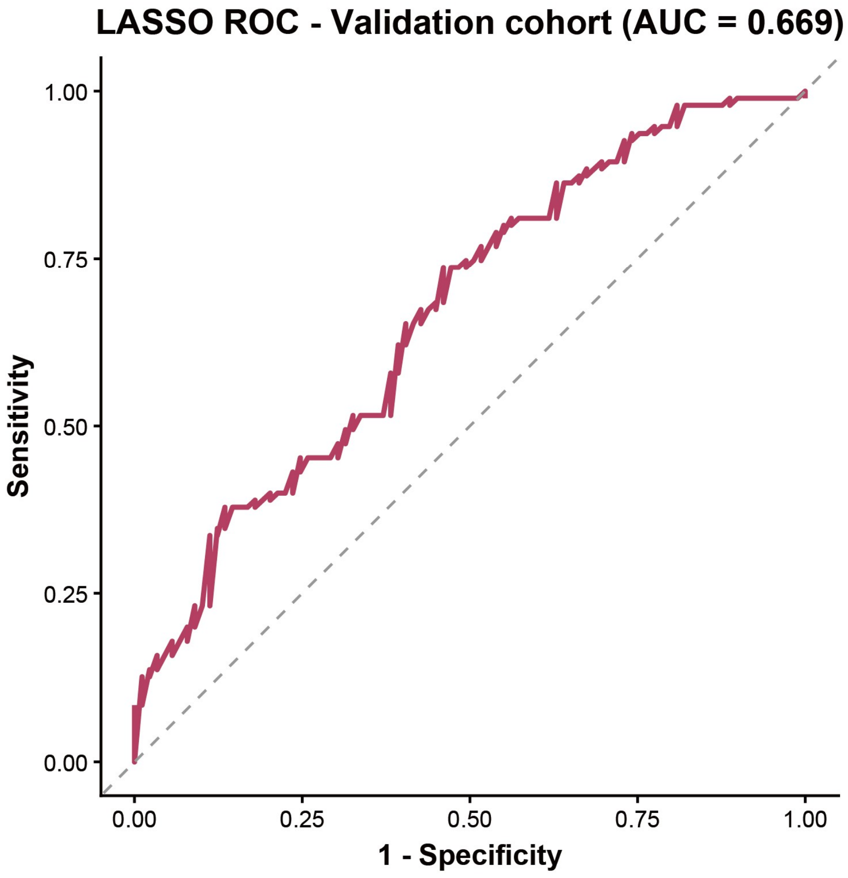
LASSO ROC - Validation cohort (AUC = 0.669)
Sensitivity
1 - Specificity
0.00
0.25
0.50
0.75
1.00

Supplementary Figure S3. Expression boxplots of CYP1B1, VEGFA, RET, FGG, and S100A9 stratified by GOLD stage (GSE47460). Control, dark blue; GOLD1, light blue; GOLD2, pale blue; GOLD3, pink; GOLD4, red. CYP1B1, VEGFA, and RET showed early alterations (GOLD1 vs. control), whereas FGG and S100A9 changed from GOLD2 onward. CYP1B1, RET, FGG, and S100A9 exhibited increasing trends across stages, while VEGFA remained persistently low, reinforcing the stage-specific relevance of the five-gene signature.

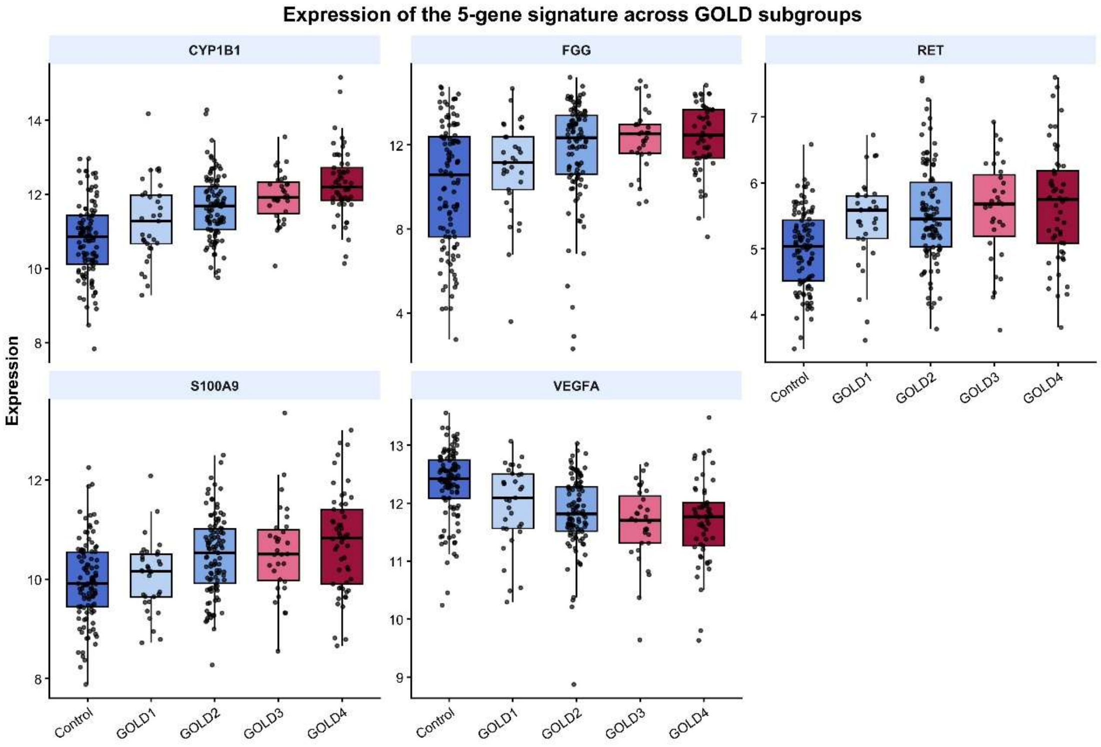

Supplementary Figure S4. Calibration curve of the five-gene nomogram for predicting COPD probability in the validation cohort. The calibration plot shows close agreement between predicted and observed outcomes, indicating good consistency and robustness of the nomogram.

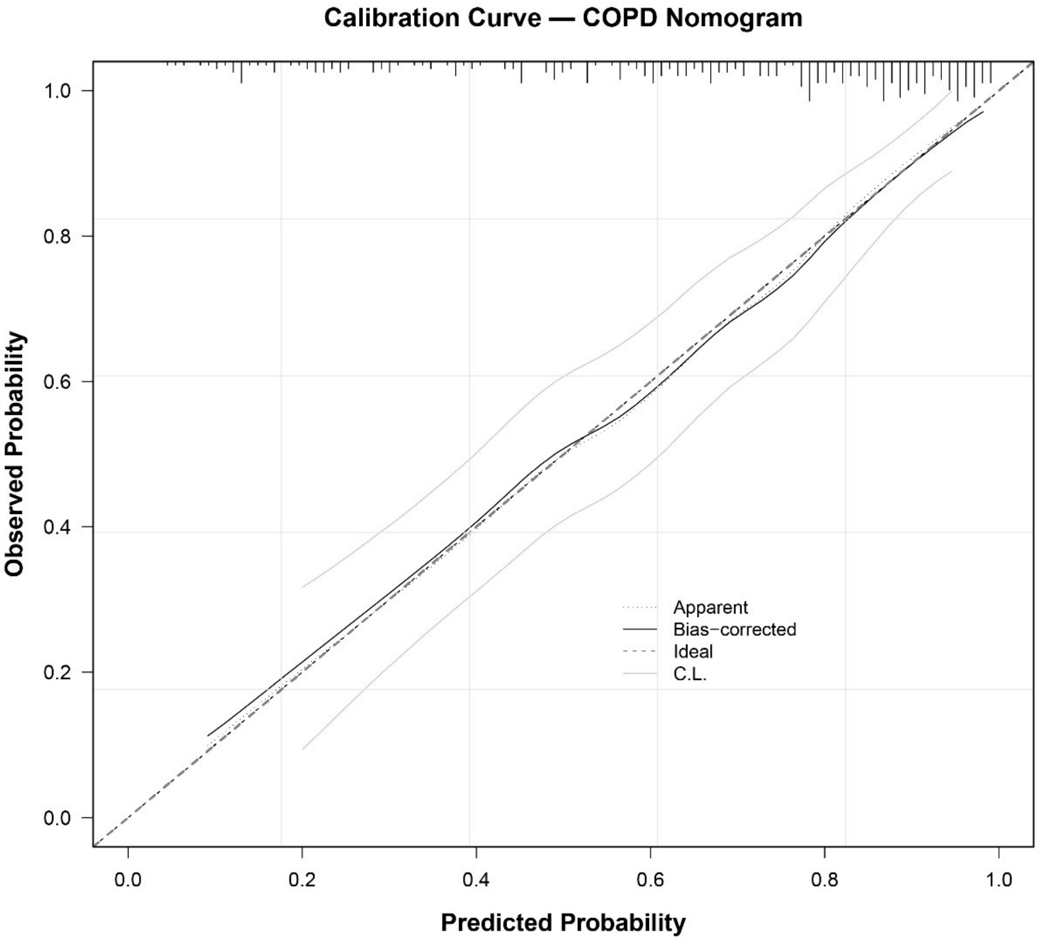

Supplementary Figure S5. Receiver operating characteristic (ROC) curves of the five individual genes (CYP1B1, VEGFA, RET, FGG, S100A9) and their combined model in the GSE20257 cohort for discriminating COPD. AUC values: CYP1B1, 0.877 (blue); VEGFA, 0.729 (orange); RET, 0.677 (green); S100A9, 0.670 (yellow); FGG, 0.49 (purple); combined five-gene model, 0.907 (bold red). The combined model outperformed all single markers, supporting panel-level robustness of the signature, although individual gene performance was heterogeneous across datasets.

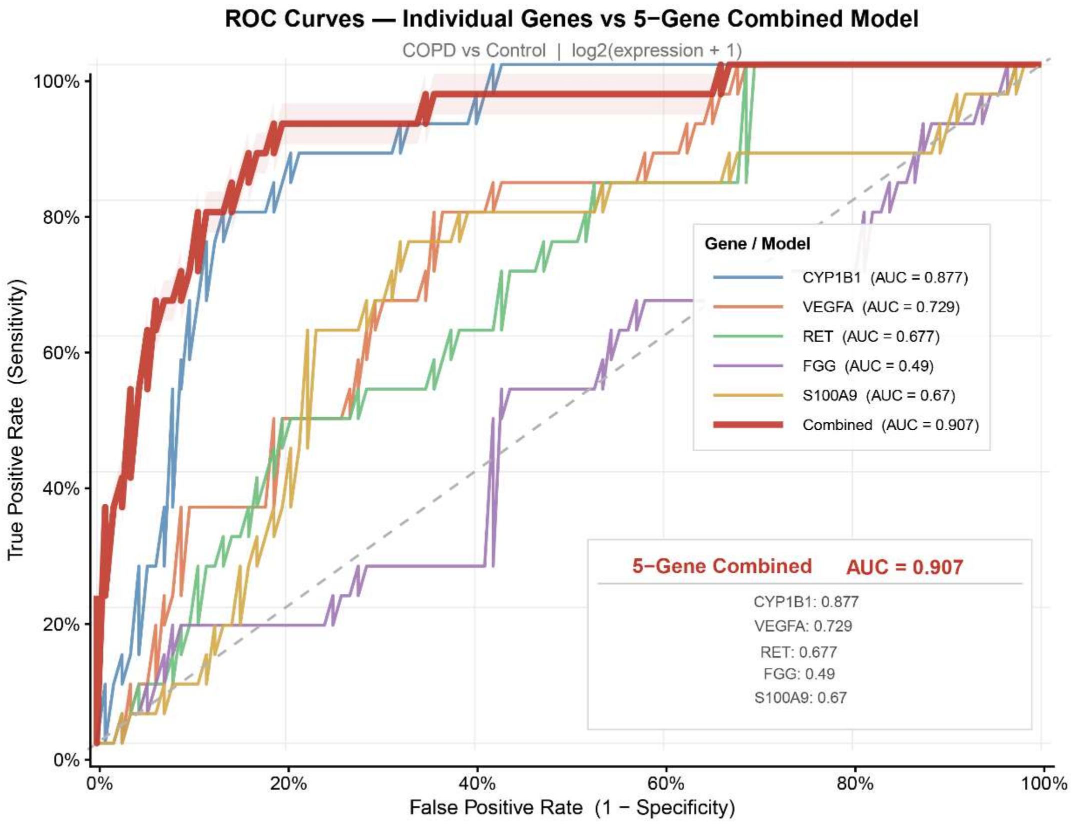

Supplementary Table S1：The full table includes all genes with a relevance score higher than the median, curated from the GeneCards database.

Supplementary Table S2: Expression statistics and differential expression analysis for the five hub genes across 15 annotated cell types in COPD versus control samples. For each gene, a separate sheet provides the number of cells (N), mean, median, and standard deviation (SD) of expression per cell type and group, as well as Wilcoxon test results (*P*-value, adjusted *P*-value, significance level) comparing COPD and control groups within each cell type. COPD, chronic obstructive pulmonary disease.

Supplementary Table S3: Candidate drugs targeting the identified hub genes, integrated from the Connectivity Map (CMap) and DSigDB databases via the Enrichr platform. A total of 178 compounds were identified. The table consists of five sheets: Drug_Ranking_Summary (overall ranking and comprehensive scores of all candidate drugs), Full_Intersection_Scored (detailed per-record CMap and DSigDB intersection results), Cmap_Filtered (CMap-only results with connectivity scores and significance), DsigDB_Filtered (DSigDB-only enrichment results), and Scoring_Method (definition and weighting of the comprehensive scoring system). Abbreviations: CMap, Connectivity Map; DSigDB, Drug Signature Database; MOA, mechanism of action; FDR, false discovery rate.
