# Supplementary figures and images for: A Synthetic Lethality-Informed Multi-Omic Framework for Identifying a Five-Gene Diagnostic Signature in Chronic Obstructive Pulmonary Disease
Source: Curr Issues Mol Biol. 2026 May 2;48(5):475. doi: 10.3390/cimb48050475 (PMC13204272; doi:10.3390/cimb48050475)

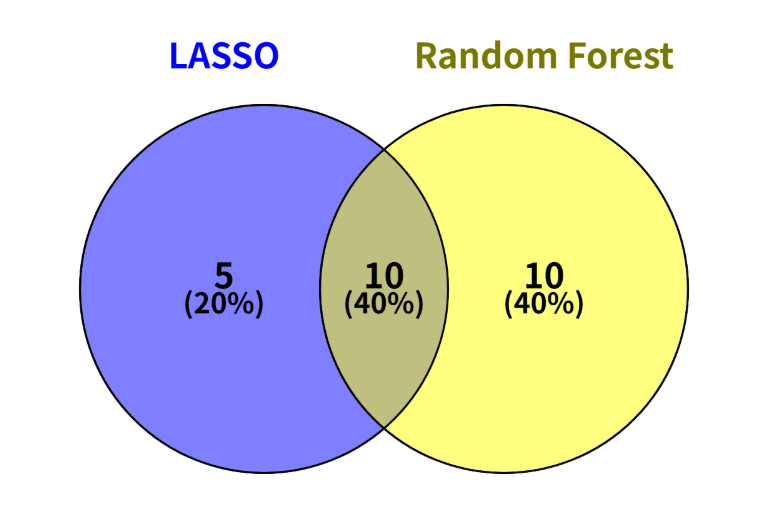

Supplement: Supplementary file 1 [file cimb-48-00475-s001.zip › Supplementary Figure S1.png]

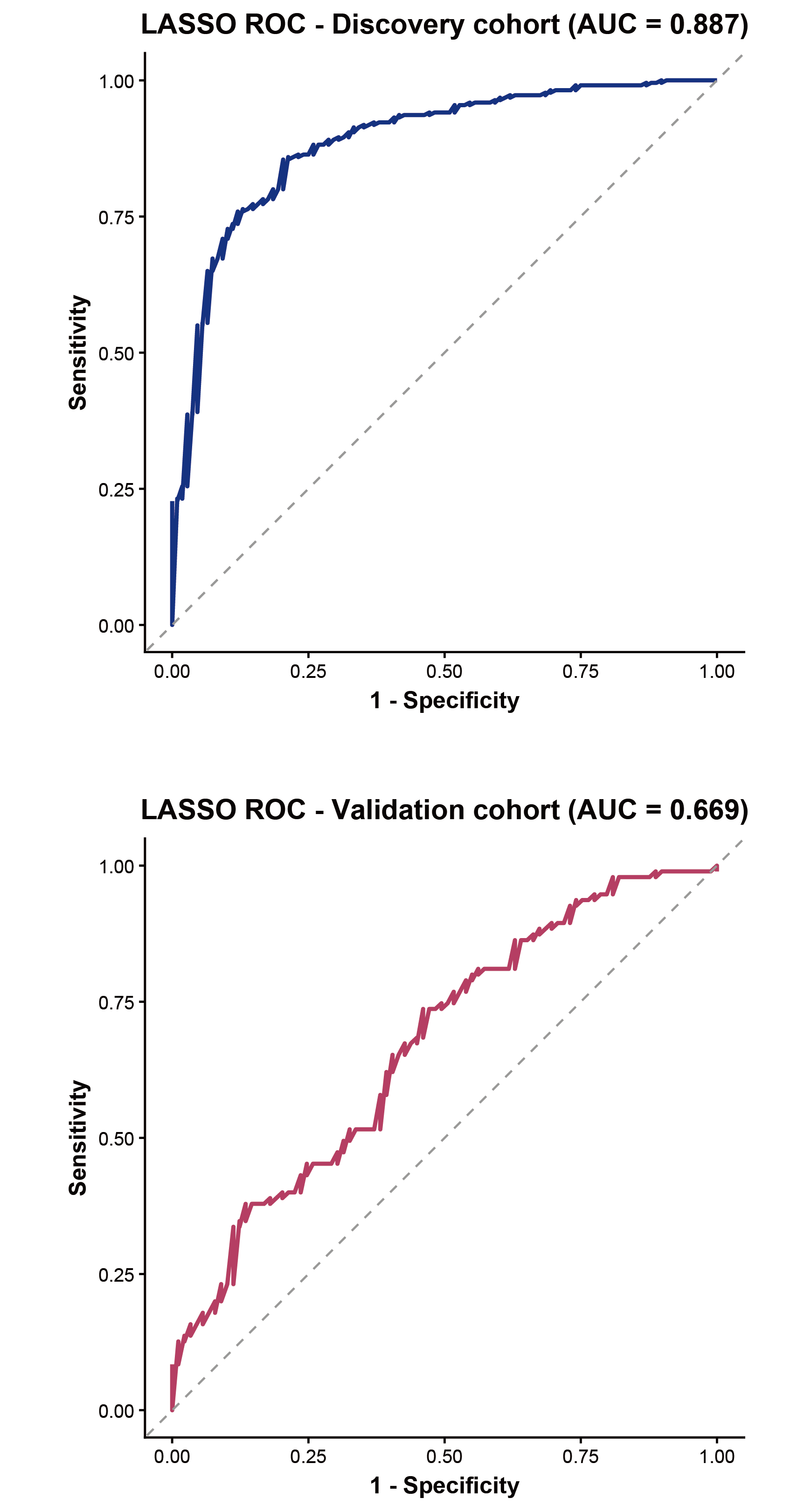

Supplement: Supplementary file 1 [file cimb-48-00475-s001.zip › Supplementary Figure S2.png]

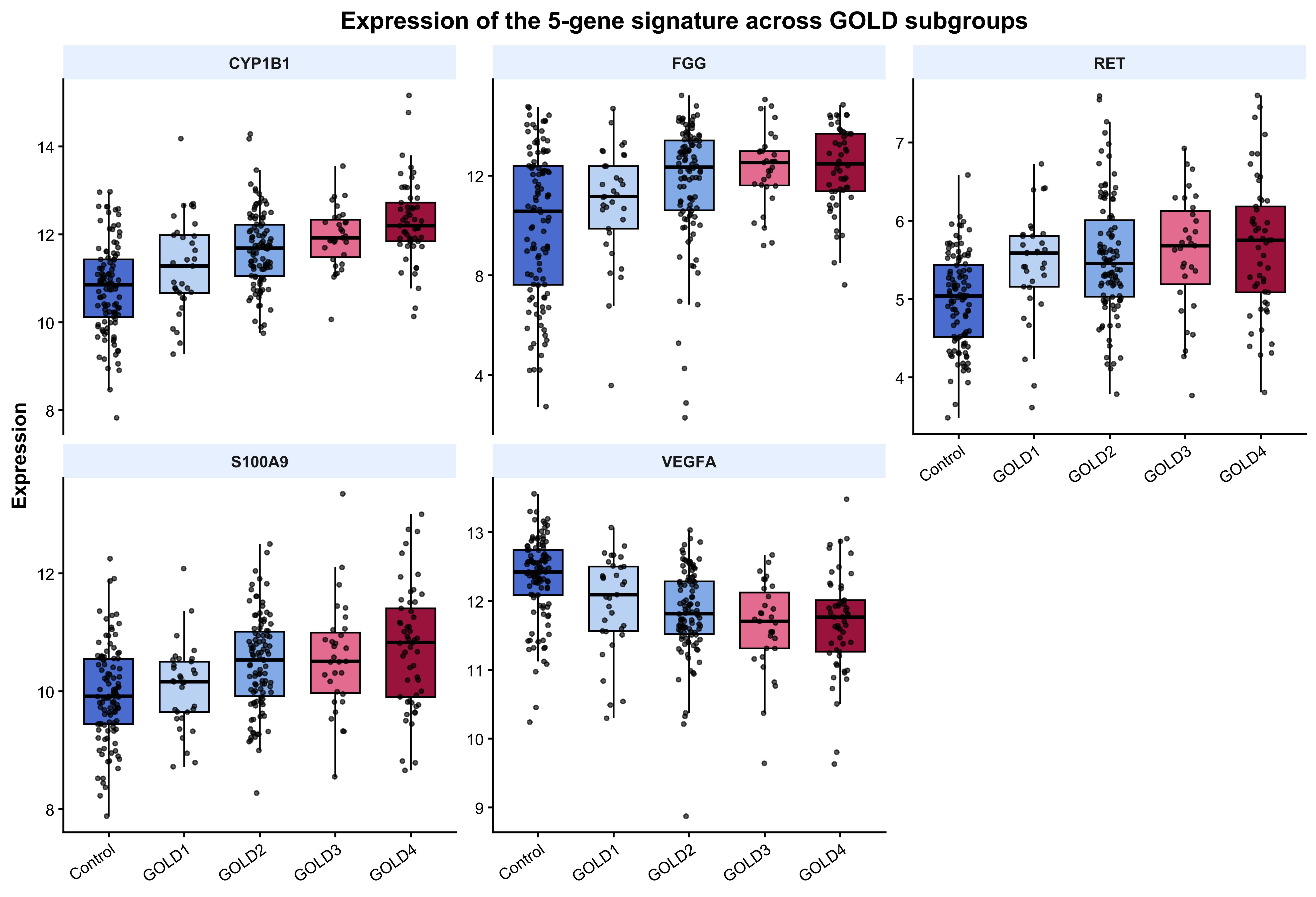

Supplement: Supplementary file 1 [file cimb-48-00475-s001.zip › Supplementary Figure S3.jpg]

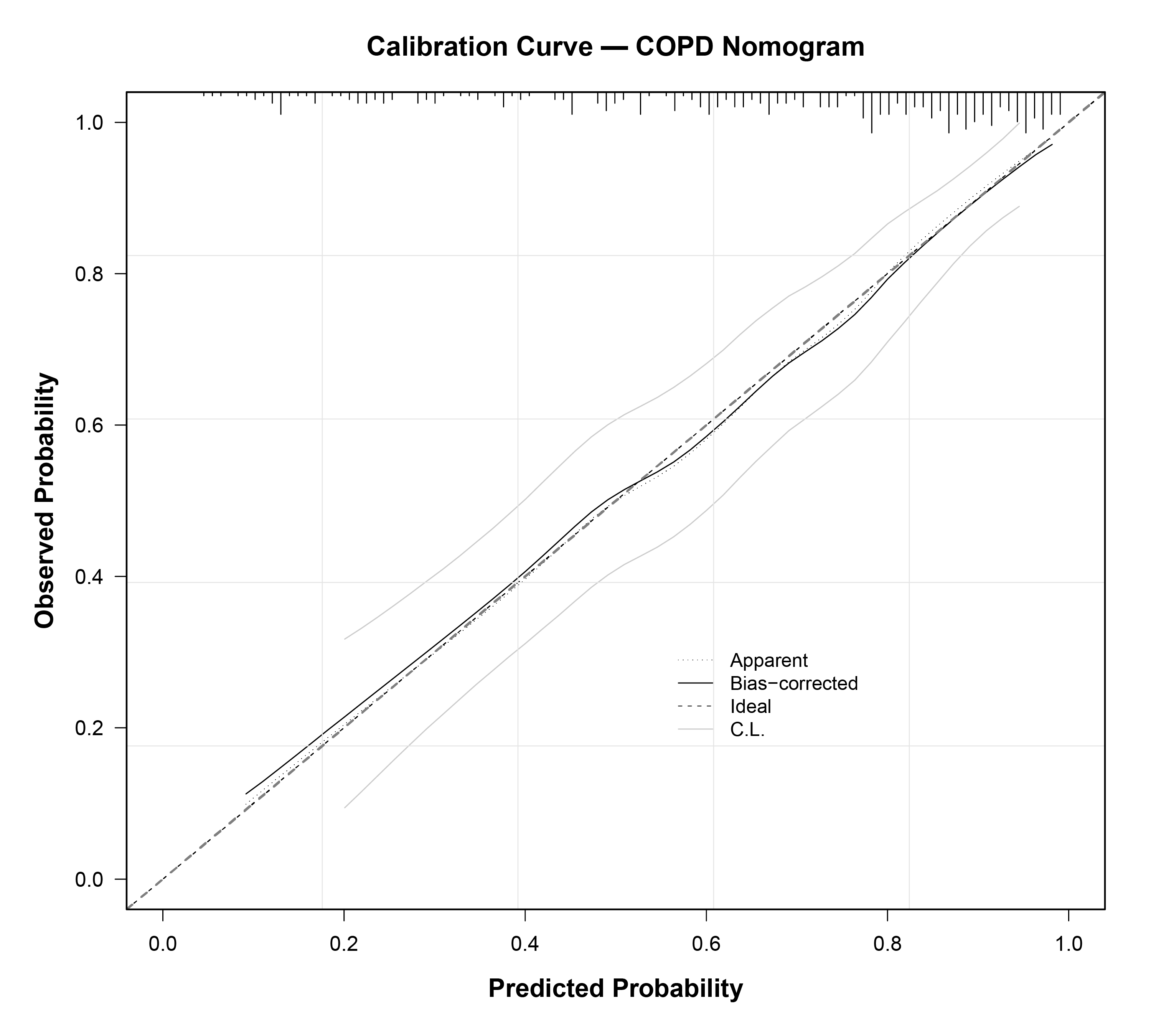

Supplement: Supplementary file 1 [file cimb-48-00475-s001.zip › Supplementary Figure S4.jpg]

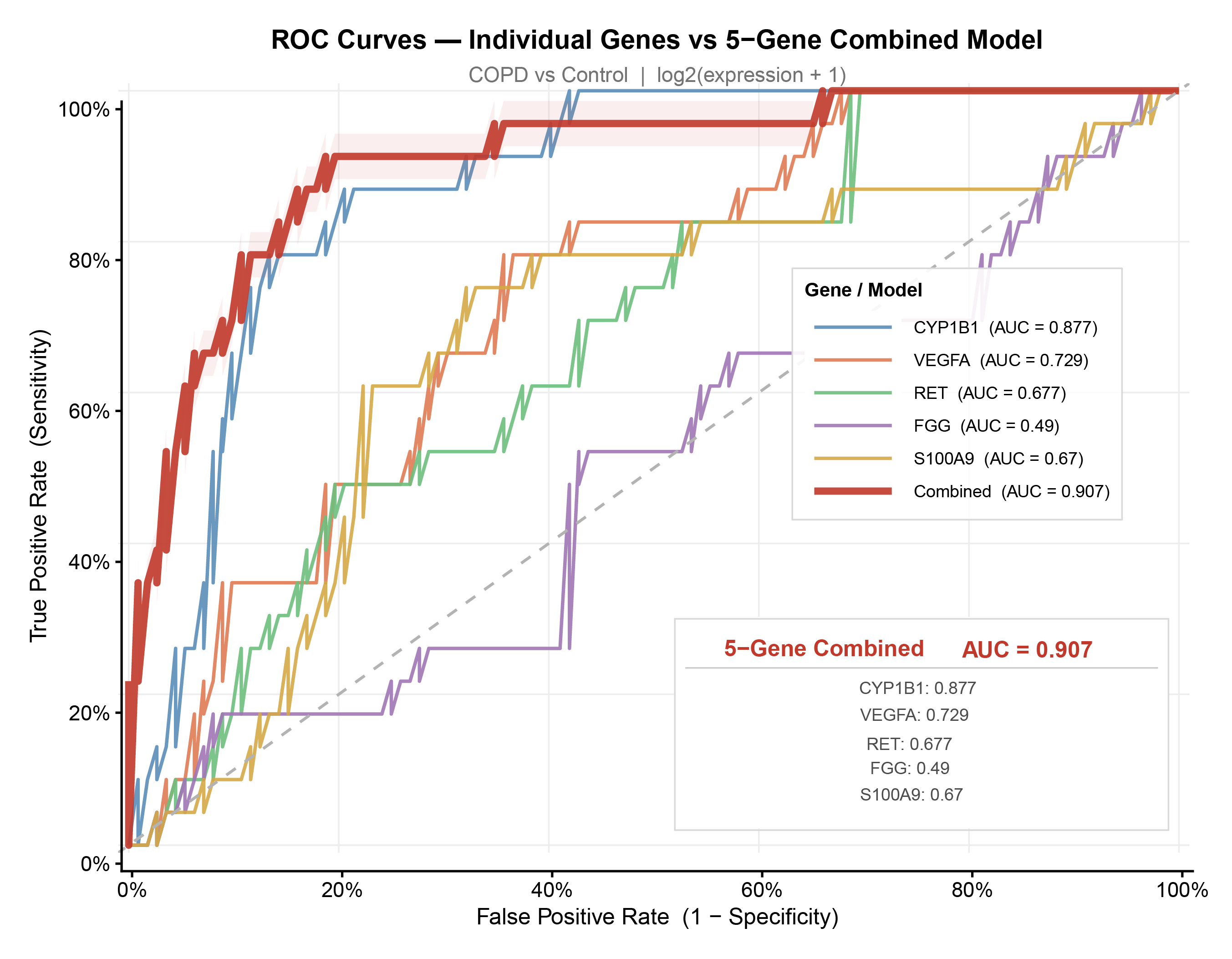

Supplement: Supplementary file 1 [file cimb-48-00475-s001.zip › Supplementary Figure S5.png]
